# Flexible Large-Area Graphene Films of 50–600 nm Thickness with High Carrier Mobility

**DOI:** 10.1007/s40820-023-01032-6

**Published:** 2023-03-03

**Authors:** Shiyu Luo, Li Peng, Yangsu Xie, Xiaoxue Cao, Xiao Wang, Xiaoting Liu, Tingting Chen, Zhanpo Han, Peidong Fan, Haiyan Sun, Ying Shen, Fan Guo, Yuxing Xia, Kaiwen Li, Xin Ming, Chao Gao

**Affiliations:** 1 MOE Key Laboratory of Macromolecular Synthesis and Functionalization, Department of Polymer Science and Engineering, Zhejiang University, Hangzhou, 310027 People’s Republic of China; 2 School of Micro-Nanoelectronics, Zhejiang University, ZJU-Hangzhou Global Scientific and Technological Innovation Center, Hangzhou, 311200 People’s Republic of China; 3 College of Chemistry and Environmental Engineering, Shenzhen University, Shenzhen, 518055 Guangdong People’s Republic of China; 4 Shenzhen Key Laboratory of Nanobiomechanics, Shenzhen Institute of Advanced Technology, Chinese Academy of Sciences, Shenzhen, 518055 People’s Republic of China; 5 Hangzhou Gaoxi Technol Co Ltd, Hangzhou, 311113 People’s Republic of China

**Keywords:** Flexible large-area graphene nanofilm, High carrier mobility, Mid-infrared detection, Electromagnetic interference shielding, Heat transfer

## Abstract

Bulk graphene nanofilms feature fast electronic and phonon transport in combination with strong light–matter interaction and thus have great potential for versatile applications, spanning from photonic, electronic, and optoelectronic devices to charge-stripping and electromagnetic shielding, etc. However, large-area flexible close-stacked graphene nanofilms with a wide thickness range have yet to be reported. Here, we report a polyacrylonitrile-assisted ‘substrate replacement’ strategy to fabricate large-area free-standing graphene oxide/polyacrylonitrile nanofilms (lateral size ~ 20 cm). Linear polyacrylonitrile chains-derived nanochannels promote the escape of gases and enable macro-assembled graphene nanofilms (nMAGs) of 50–600 nm thickness following heat treatment at 3,000 °C. The uniform nMAGs exhibit 802–1,540 cm^2^ V^−1^ s^−1^ carrier mobility, 4.3–4.7 ps carrier lifetime, and > 1,581 W m^−1^ K^−1^ thermal conductivity (nMAG-assembled 10 µm-thick films, mMAGs). nMAGs are highly flexible and show no structure damage even after 1.0 × 10^5^ cycles of folding–unfolding. Furthermore, nMAGs broaden the detection region of graphene/silicon heterojunction from near-infrared to mid-infrared and demonstrate higher absolute electromagnetic interference (EMI) shielding effectiveness than state-of-the-art EMI materials of the same thickness. These results are expected to lead to the broad applications of such bulk nanofilms, especially as micro/nanoelectronic and optoelectronic platforms.

**Supplementary Information:**

The online version contains supplementary material available at 10.1007/s40820-023-01032-6.

## Highlights

- Large-area production of self-standing graphene nanofilm (~ 20 cm) through a clean ‘substrate replacement’ strategy.
- Realizing highly crystalline graphene nanofilms without micro-gasbags by introducing polymers.
- The graphene nanofilms demonstrate a solid light–matter interaction (photoelectric conversion in the mid-infrared and electromagnetic interference (EMI) shielding in X-band) with performance beyond state-of-the-art graphene/silicon diodes and EMI materials.

**Supplementary Information:**

The online version contains supplementary material available at 10.1007/s40820-023-01032-6.

## Graphical abstract

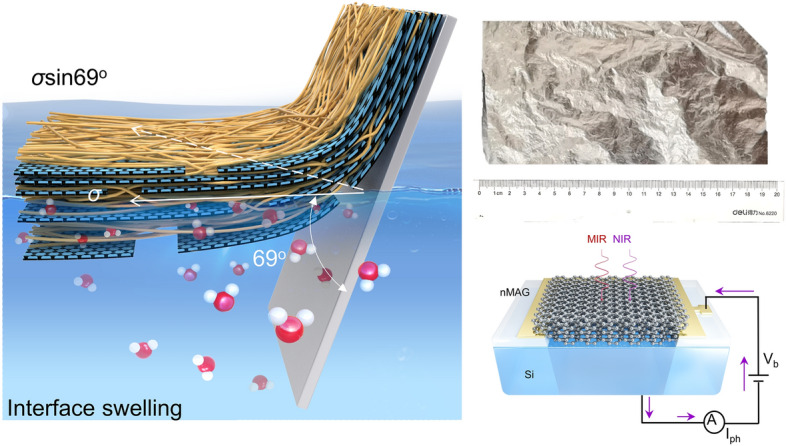

## Introduction

Bulk two-dimensional (2D) nanomaterials with close-stacked structure have attracted tremendous interest since they combine monolayer-like properties with robust performances arising from their bulk nature, including high light absorption/reflection, long carrier lifetime and free path, and strong coulomb scattering [1], etc*.* In particular, highly crystalline and close-stacked graphene nanofilms demonstrate outstanding potential in the fields of photonic, electronic, and optoelectronic devices, such as terahertz plasmons [2], wireless antennas for 5G and 6G communication [3], radiofrequency transistors [4], and mid-infrared to X-ray detection [5], with performances beyond those anticipated from their monolayer and few-layer counterparts. Especially the broad range of thickness extends the applications of graphene nanofilms to charge-stripping accumulation of light/heavy ions in ion cyclotrons [6], electromagnetic interference shielding with ultra-lightweight in stealth technology [7], etc. Besides, graphene nanofilms serve as a hub to explore the relationship between individual assembly units and the corresponding macro-material formed therefrom, connecting the nano with the macro-world. Nevertheless, the production of large-area crystalline close-stacked graphene nanofilms in a wide thickness range has yet to be realized.

There are two strategies for large-area fabrication of graphene nanofilms: chemical vapor deposition (CVD) and wet assembly. The nickel catalytic CVD strategy is a general approach to synthesizing bulk graphite nanofilm with tens to hundreds of nanometers thick [8, 9], which is always accompanied by a contaminative separation process from etchant and transfer agent. Meantime, the structural defects in CVD-grown graphite nanofilms, such as edges, stacking disorders, heterogeneous thickness, and wrinkles, are challenging to remove due to the carbon diffusion mechanism, significantly lowering their electrical and thermal conducting performances. Zhang et al. reported a single-crystal graphite film (about 3 cm) with an improved CVD strategy [10]. Nevertheless, the thickness uniformity in the nanoscale is still a challenge. Besides, the micron-scale thickness restricts their back-end-of-line integration with electronic devices due to their weak interfacial adhesion and long electron and phonon migration paths.

Recently, our group reported a simple, clean, bottom-up wet assembly method to rapidly synthesize uniform graphene nanofilms based on self-supporting graphene oxide (GO) nanofilms [2]. The 2D conformation of GO endows the GO nanofilms with high strength and stability, which helps in keeping the self-standing state of nMAGs during the thermal annealing process. However, the brittle substrate used limited the lateral size of these films to < 4.2 cm in diameter. Besides, the thickness of graphene nanofilms obtained is in the range of 16–48 nm. At thicknesses > 50 nm, micro-gasbags are generated in nMAGs due to the hindered gas escape during the annealing process resulting in wrinkled surfaces [11].

Here, starting from GO/polyacrylonitrile (PAN) films, we propose a ‘substrate replacement’ strategy to fabricate large-size (20 cm) and close-stacked graphene films in a wide thickness range of 50–600 nm. The facile separation at the interface and free gas escape during annealing are attributed to the introduction of PAN, which enables complete transformation into intact graphene lattices. The nMAGs show good foldability (1.0 × 10^5^ cycles) and carrier mobility of 1,540 cm^2^ V^−1^ s^−1^ (electrical conductivity, 2.04 MS m^−1^). As a demonstration, nMAGs achieve the minimum commercial electromagnetic interference (EMI) shielding effectiveness of 20 dB at thickness < 100 nm. The strong photo-thermionic (PTI) emission effect endows the nMAG/silicon diode with a broad response wavelength of 1.8–4 µm. Furthermore, by layer-by-layer assembly of 200-nm-thick nMAGs, we fabricated 10-µm-thick graphene films, which show low wrinkle density and thus higher thermal conductivity (1581 W m^−1^ K^−1^) than commercial artificial graphite films with the same thickness.

## Experimental Section

### Materials

Graphene oxide (GO, 28 μm average platelet size, Fig. S1) dispersions were purchased from Hangzhou Gaoxi Technology Co., Ltd. (http://www.gaoxitech.com/). Polyacrylonitrile (PAN, Molecular weight 80,000) was purchased from Sigma-Aldrich. Other reagents were purchased from Sinopharm Chemical Reagent Co., Ltd and used as received.

### Preparation of nMAG

Figure S2 illustrates the preparation process of GO/PAN composite film. 10 g of PAN was dissolved in 90 g of DMF to get a (10 wt%) solution. The PAN content in the film was varied to be either 50, 60, or 70 wt% to study its effect on the graphitization degree of the composite film [12]. The PAN solution (10 wt%) was mixed with GO dispersion (13 mg g^−1^, Fig. S1) and DMF to obtain a solution with a solid weight of 1%. GO/PAN films with different thicknesses were made by the Mayer rod coating method described [13]. After drying, the GO/PAN film (only the edge was cut) with the substrate was slowly immersed in water at an inclination angle range of 90–180° with the substrate facing the water. The film gradually separated from the plate due to the water surface tension and finally floated on the water surface. The solvent was then exchanged for ethyl alcohol, and a rough graphite plate was used to obtain a free-standing film. After drying in an oven, the free-standing GO/PAN film was heated at 270 °C in the air for 1.5 h for pre-oxidation, followed by thermal annealing at 3,000 °C to restore its bulk structure.

### Preparation of mMAG and GPF

**mMAG**: The precursor of mMAG was prepared by layer-by-layer assembly of GO/PAN nanofilm. First, the Mayer rod coating method was used to spread the GO/PAN solution on the quartz plate. After drying, PVA aqueous solution (0.05 wt%) was sprayed on the surface of the GO/PAN layer. A new GO/PAN layer was then deposited on the PVA layer. These processes were repeated until the required thickness was reached.

**GPF**: The precursor of GPF (GO/PAN film) was prepared through doctor blade coating, widely used to produce thin films on large-area surfaces [12]. A doctor blade spread the GO/PAN solution (1–2 wt%) directly on the quartz plate. After drying at 60 °C, a 20-μm-thick GO/PAN film was obtained.

These two films above were then heated at 270 °C in the air for 1.5 h for pre-oxidation, followed by thermal annealing at 3000 °C to restore the structure. Finally, the 10-μm-thick mMAG and GPF were obtained.

### Preparation of nMAG-Based Infrared Devices

The device was fabricated on a lightly doped n-type silicon wafer. (1) nMAG (50 nm) was transferred onto the silicon window. The gap between nMAG and silicon was filled with deionized water to unfold the wrinkles. The surface of nMAG was then purged with nitrogen before the solvent was entirely volatilized. (2) The device was further thermally annealed at 600 °C in N_2_ to enhance the interface binding between nMAG and the silicon wafer. (3) Finally, e-beam deposition and thermal evaporation (Angstrom Engineering) were used to deposit Cr/Au (5/100 nm) as top electrodes. Ohmic contact was formed between the back side of the silicon and copper tape. Au wires were then bonded to the top and back electrodes [5].

### Infrared Photoresponse Measurement

The photoresponse was measured using lock-in and current amplifiers Stanford SR830 and SR560. Periodic pulse lasers (Light Conversion, OPA-Series, 1 to 10 μm, 200 fs pulse width, and 100 kHz repetition rate) were used as the light source. The laser beams were focused by a reflection objective and finally onto the device. Direct-current electrical characterizations were performed using a Keithley 2460 dual-channel digital source meter. A 1 GHz bandwidth oscilloscope was used to measure the transient response [5].

### Electromagnetic Interference Shielding Performance Measurement

EMI shielding performances were measured by a vector network analyzer (ZNB: 40, Rohde & Schwarz, Germany) with the waveguide method (TT-EMWG-X, 8.2–12.4 GHz). The nMAG sample was transferred to the PI film (Fig. S19) for shielding performance measurement and clamped by the holder (length = 22.86 mm, width = 10.16 mm). To reduce contact resistance, the holders should be tightly connected. Scattering parameters (S_11_, S_22_, S_12_, S_21_) obtained by ZNB 40 were used to calculate the EMI SE.

The EMI shielding performance was calculated based on S parameters (S_11_ and S_21_); the transmission power (T; T =|S_21_|^2^), reflectivity power (R; R =|S_11_|^2^), and absorption power (A; A + R + T = 1) [14, 15].

The total EMI shielding effectiveness (*SE*_T_), reflection effectiveness (*SE*_R_), and absorption effectiveness (*SE*_A_) were calculated as follows.

1 $$SE_{\rm T}=10\times log_{10}{\left(\frac{1}{\mathrm{T}}\right)}$$

2 $$SE_{\rm R}=10\times log_{10}{\left(\frac{1}{1-\mathrm{R}}\right)}$$

3 $$SE_{\rm A}=10\times log_{10}{\left(\frac{1-\mathrm{R}}{\mathrm{T}}\right)}$$

The influence of the PI substrate should be subtracted from the final result.

### Characterization

Scanning electron microscopy (SEM) images were taken on a Hitachi S4800 field emission system at the acceleration voltage of 3 kV. X-ray diffraction (XRD) was measured on an X’Pert PRO diffractometer (PANalytical) using Cu Kα1 radiation with an X-ray wavelength of 1.5406 Å. Raman spectroscopy was performed using a confocal Raman microscope (Senterra, BRUKER) and a Renishaw Invia system with a 532-nm laser. A focused ion beam (Helios 450HP FIB) was used to prepare cross-sectional samples for transmission electron microscopy (TEM) examination. TEM images were acquired on a Hitachi H-9500 instrument operating at 300 kV. X-ray photoelectron spectroscopy (XPS) was performed with a PHI 5000C ESCA System operated at 14.0 kV, and all binding energies were referenced to the C 1* s* neutral carbon peak at 284.6 eV. Scanning tunneling microscopy (STM) experiments were carried out in an ultrahigh vacuum (UHV) low-temperature (~ 77 K) STM system (UNISOKU USM-1500S). The STM topography was typically taken with a sample bias *V* =  − 100 mV and a setpoint current *I* = 200 pA. Scanning transmission electron microscopy (STEM) and energy-dispersive X-ray mapping (EDX) were taken using a spherical aberration-corrected transmission electron microscope (Titan Chemi STEM). Wide-angle X-ray scattering (WAXS) and small-angle X-ray scattering (SAXS) measurements were carried out in the 14b beamline of the Shanghai Synchrotron Radiation Facility in Shanghai, Republic of China. Thin-film samples were transferred to silicon wafer substrates for measurement. The water contact angle was measured by a contact angle and surface tension measuring instrument (1008360Q). Thermogravimetric Analysis (TGA) was carried out in air or nitrogen at the heating rate of 10 °C min^−1^ (Perkin-Elmer Pyris). Electron mobilities were determined using a Hall effect measurement system (Lakeshore 7604). The time-of-flight secondary ion mass spectrometry (ToF–SIMS) characterization was tested by PHI nanoTOF II Time-of-Flight SIMS equipped with GCIB Gun to sputter. The Fourier transform infrared (FTIR) spectrometer was tested by Nicolet 6700.

The electrical properties, such as temperature coefficient of resistance, were measured using a current source (Keithley 6221) and an oscilloscope (Tektronix DPO 3052 Digital Phosphor Oscilloscope). A Janis closed cycle refrigerator (CCR) system was employed to provide a stable and reliable environmental temperature from 320 to 7 K. Hall mobility was tested on a Nanometrics HL5500 Hall system using a van der Pauw configuration at room temperature. Thermal conductivity was measured using Netzsch NanoFlash LFA 467 instrument.

## Results and Discussion

### Preparation of Ultrathin Free-standing GO/PAN Film

**Fig. 1:**
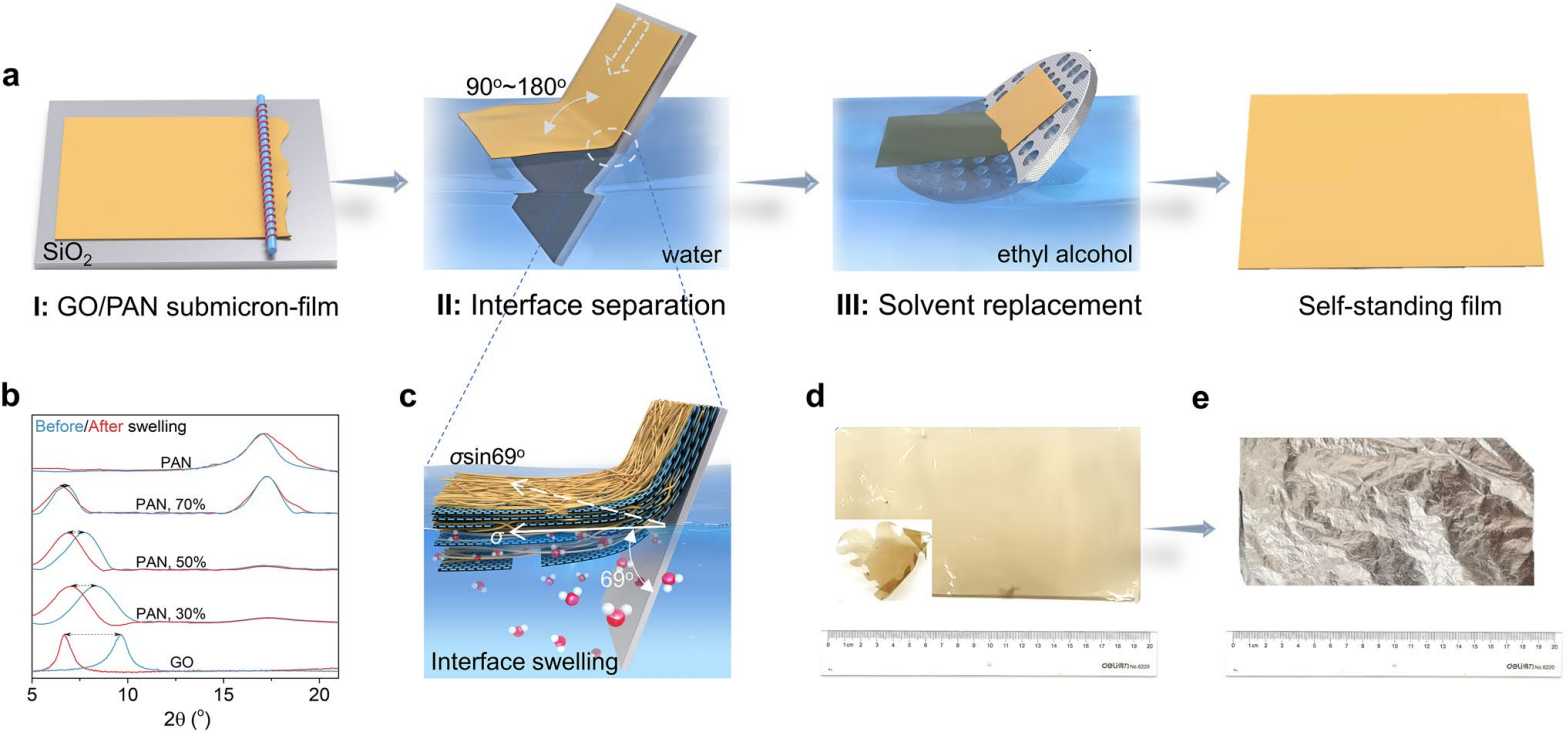
Preparation of ultrathin free-standing GO/PAN film. **a** Preparation process of a self-standing GO/PAN film. **b** XRD spectra of GO/PAN films with different mass ratios before and after swelling in water. **c** Detachment of GO/PAN film from the quartz substrate on the water surface (Step II). **d** Images of free-standing GO/PAN film (190 nm thick) with a large area and having the ‘Qiushi eagle’ shape (corner). **e** Free-standing nMAG (100 nm thick) treated under argon atmosphere at 3,000 °C for 1 h

Figure 1a illustrates the three stages in preparing ultrathin GO/PAN films (Fig. S2). GO/PAN films were deposited on quartz (SiO_2_) surfaces by the Mayer rod coating technique (Stage I). Driven by the surface tension (*σ*) of water, the film was detached from the SiO_2_ substrate (Fig. S3a), leaving a water-supported GO/PAN film (Stage II). The water contact angle (marked as *θ*) of the substrate and swelling property of the GO/PAN film determine the separation process at the interface (Fig. 1c). The high water contact angle of the quartz plate (69°, Fig. S3b) ensures that most surface tension (*σ*sin*θ*) falls in the vertical direction of the substrate [16]. The combination of soluble and insoluble components in GO/PAN film promotes synergistically reducing interfacial binding energy between the nanofilm and quartz substrate. The excellent dispersion of GO sheets facilitates the swelling of the GO/PAN film when contacted with water, weakening the interfacial binding energy between GO/PAN and substrate. The insoluble PAN molecular chains act as physical cross-linking agents, ensuring the integrity of the GO/PAN film in the subsequent interface separation step. As shown in XRD patterns (Fig. 1b), with the increase in PAN content from 0 to 70%, the variation in *d*-spacing of the GO/PAN film before and after the swelling process is reduced from 0.39 to 0.07 nm (Fig. S3c).

Next, we transferred the GO/PAN film from the surface of the water to ethyl alcohol solvent using a porous and rough graphite plate (Stage III). Due to the volatile nature of ethanol and its low surface tension, the composite film has low adhesion to the rough substrate. After complete evaporation of the solvent, the GO/PAN film spontaneously detached from the substrate, leaving a free-standing GO/PAN nanofilm with a lateral size of 20 cm (Fig. 1d). The shape of the GO/PAN films can be adjusted to any form, such as round, square, or to even complex shapes, such as a ‘Qiushi eagle’ (Figs. 1d and S4a). After heat treatment at 3000 °C, free-standing graphene nanofilms (Figs. 1e and S4b, c) with high crystallinity were obtained.

It is worth mentioning that this route circumvents the strict requirements on the strength of the nanofilm and substrate structure while avoiding etchants and transfer agents (contaminants) usually used in conventional bottom-up methods [17]. In principle, increasing the mold size allows for obtaining free-standing GO/PAN nanofilms of larger sizes, and thus, many large-area self-standing nanofilms based on 2D materials or polymers with a large area could be prepared in a wide range of thickness. For example, following the GO/PAN system protocol, we successfully prepared self-supporting rGO (GO reduced by hydroiodic acid, HI)/sodium–lignin–sulfonate composite nanofilms by substrate replacement strategy (Table S2). Unlike the GO/PAN system, in the rGO/sodium–lignin–sulfonate system, rGO sheets act as the hydrophobic physical cross-linking agent, and the polymer acts as the hydrophilic part.

### PAN-derived Gas Escape Channels

**Fig. 2:**
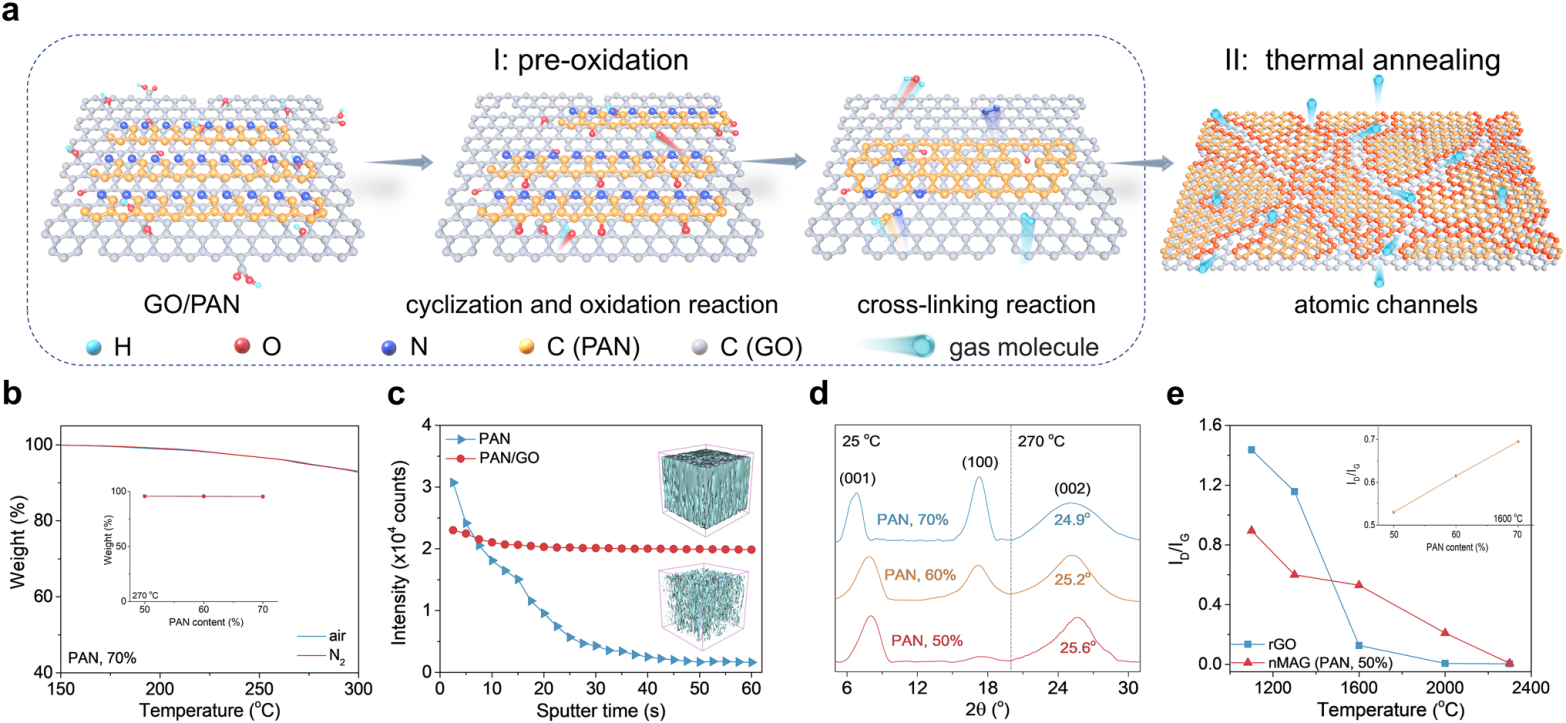
PAN-derived atomic-scale gas escape channels. **a** Schematic diagram of the formation of atomic channels in GO/PAN. **b** TGA plot of GO/PAN (PAN, 70%) in N_2_ and air atmosphere; inset shows the residual masses for nMAGs with different PAN contents heated to 270 °C in N_2_ and air. **c** ToF–SIMS elemental depth profiles for oxygen element in 600-nm-thick pre-oxidized GO/PAN film (PAN, 70%) and PAN film, and their corresponding 3D mapping images of oxygen (inset). **d** X-ray diffraction patterns of GO/PAN films with different PAN contents before and after pre-oxidation at 270 °C. **e** *I*_D_/*I*_G_ values of nMAG (PAN, 50%) and rGO heat treated at different temperatures. The inset shows *I*_D_/*I*_G_ values of 1,600 °C-treated nMAGs assembled with varying contents of PAN

Apart from cross-linking 2D GO sheets, derivatives of PAN can create channels for eliminating gases that evolved during the heat treatment (Fig. 2a). For PAN, the pre-oxidation stage at 180–300 °C is a prerequisite to forming high-quality carbonaceous structures [18]. In the present case, the pre-oxidation process of PAN occurs in a closed system with no external oxygen input. Thermogravimetric analysis (TGA, Figs. 2b and S5) plots show that PAN/GO systems have almost identical weight loss irrespective of the heating atmosphere, indicating that the GO/PAN system pre-oxidation occurs through self-oxygenation without external oxygen input [19]. A possible reason is that the dense 2D planar structure of GO and the high inner pressure caused by the release of gases inside the material prevent oxygen penetration from the external source [15]. The uniform dispersion of GO sheets and self-oxygenation effect endow the pre-oxidized GO/PAN system with a uniform oxygen distribution in a 3D structure (100 μm × 100 μm × 300 nm) with a stable oxygen intensity of 2 × 10^4^ in time-of-flight secondary ion mass spectrometry (ToF–SIMS, Fig. 2c). In contrast, in the pre-oxidized PAN film, the oxygen intensity varies rapidly from 3.07 × 10^4^ (surface) to 0.16 × 10^4^ (core), indicating the formation of a ‘skin–core structure’ (Figs. 2c and S6) [20].

During the self-oxidation (180–300 °C) process [21–23], in the presence of GO, PAN evolves from a disordered organic macromolecule into an ordered 2D structure (Fig. 2a). In the pre-oxidation process (stage I), PAN undergoes cyclization reactions by free radicals. The electronegative functional groups on the GO surface, such as carboxyl, accelerate the cyclization reaction by nucleophilic attack on the nitrile group of the PAN chains [24]. Besides, oxygen-containing free radicals generated by the decomposition of GO during the cyclization process participate in the dehydrogenation and oxidation processes, further oxidizing the cyclized PAN chains and forming a carbonyl oxygen structure [24, 25]. This process was proved by the faded C-O (1,050 cm^−1^ in FTIR, 286 eV in XPS) and C–OH (1385 cm^−1^) groups in GO and the emerged C = O (1,730 cm^−1^) group after 270 °C treatment (Figs. S7 and S8). The ladder structure then evolves into a cross-linked 2D structure accompanied by dehydrogenation reactions [26]. The merged (002) peaks at around 25°, the faded (100) PAN peak at 17°, and the (001) peak at about 10° in the XRD of PAN/GO before and after 270 °C pre-oxidation further confirm the reduction of GO and the structural transformation of PAN (Fig. S9). The graphite-like (002) peak indicates the emergence of a graphene-like 2D cross-linked structure. With the increase in PAN content from 50 to 70%, the cross-linked structure becomes more disordered, increasing the full width at half maximum (FWHM) of the (002) peak from 4.25° to 5.0° and 8.5° and shifting the (002) peak from 25.6° to 25.2° and 24.9° (Fig. 2d). After 3,000 °C treatment, due to the complete graphitization, no peaks of oxygen-containing groups in the FTIR and XPS spectra (Figs. S7 and S8) and no obvious defects (D peak, 1,350 cm^−1^) in Raman were found in nMAGs.

Compared to the GO sheets, the 2D cross-linked graphene structures have more edges and atomic defects in the subsequent high-temperature treatment (stage II, Fig. 2a). As shown in Figs. 2e and S10, the *I*_D_/*I*_G_ and (O + N)/C values of nMAG (PAN, 50%) are higher than observed for rGO when the annealing temperature exceeds 1,300 °C (Fig. 2e). This result indicates that PAN-derived 2D cross-linked graphene structures have a higher defect content than rGO treated at the same temperature, suggesting the presence of a more significant number of atomic-scale channels for gas escape in GO/PAN-based nMAGs. The advantage of nanochannels in the GO/PAN system is maintained up to 2300 °C. With the increase in PAN content, the defects of nMAGs (taking 1600 °C-treated nMAGs as examples, inset of Fig. 2e) increase. Therefore, a higher PAN content can further promote the escape of gases and inhibit the formation of gasbags at higher thicknesses.

### Structure and Flexibility of nMAG

**Fig. 3:**
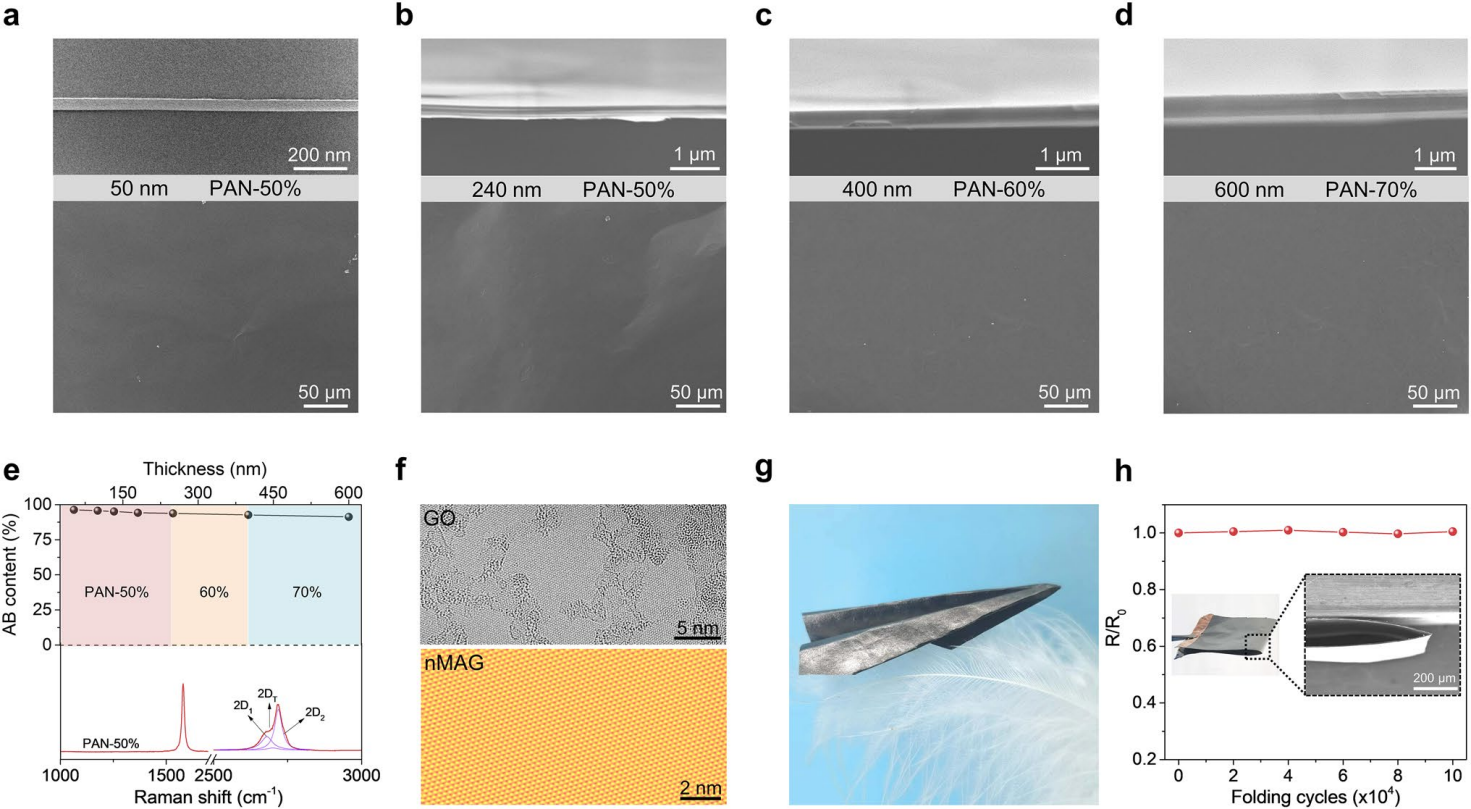
Structure and flexibility of nMAGs. **a** HR-TEM (cross-section, Up) and SEM (surface, Down) images of 50 nm-thick nMAG. **b**–**d** Cross-sectional and surface SEM images of nMAGs with different thicknesses. **e** AB content of nMAGs with different thicknesses (Up) and Raman spectrum of a 50-nm-thick nMAG (Down). **f** HR-TEM image of GO (Up) and STM topography image of nMAG (Down). **g** Paper plane folded with 600-nm-thick nMAG. **h** Resistance variation of 600-nm-thick nMAG after repeated folding

As shown in Fig. 3a, a dense cross-section and a smooth surface morphology are seen for nMAGs after 3,000 °C treatment in the 50–240 nm thickness range with a PAN content of 50% in the raw films. With increasing thickness of nMAGs, a rough surface with micro-gasbags is observed (Fig. S11a). As the proportion of PAN is increased from 50 to 60% and 70%, close-packed nMAGs are obtained up to thicknesses from 240 to 400 and 600 nm, respectively (Figs. 3b-d and S11b, c). In contrast, a pure graphene nanofilm with fewer defects generates gasbags at a thickness above 50 nm during 3,000 °C treatment (Fig. S12).

The 2D topology template of GO has a solid directional effect during carbonization and graphitization of PAN under high-temperature annealing [12, 19], resulting in oriented graphene layers parallel to the thin films and giving rise to a high crystallinity (Fig. S13). As shown in Fig. 3e, the tiny 2D_T_ peak (2,700 cm^−1^) in the Raman spectrum indicates an AB content of about 96% determined from the I(2D_T_)/I(2D_T_ + 2D_2_) value at 50 nm thickness [27]. The high degree of graphitization is consistent with the separated (101) peak and the strong (112) peak in the wide-angle X-ray scattering (transmission mode) spectrum (Fig. S14). The observed graphite-like *d*-spacing of 3.35 Å for the (002) peak (2θ, 26.5°) in XRD further confirms this result (Fig. S15). In addition, with the increase in thickness and PAN content, the GO-induced graphitization effect is suppressed, bringing more edge defects and stacking disorders to the nMAGs. Consequently, the AB content of nMAGs is reduced from 96% (50 nm thick) to 91% (600 nm thick, Fig. 3e).

The graphene lattices of nMAG before and after 3,000 °C annealing were visualized at atomic resolution to evaluate the extent of structural restoration. As shown in Figs. 3f and S16, clean and intact hexagonal lattice areas 6–15 nm in diameter and the surrounding defects were identified by HR-TEM. After annealing, a perfect long-range triangular lattice structure is seen in STM image, indicating a complete structural restoration to graphene with AB stacking geometry. The nanoscale thickness endows nMAG with good flexibility. They can endure complex folding like an airplane (Fig. 3g). Furthermore, there is little change in resistance after 1.0 × 10^5^ folding (Fig. 3h), and no apparent damages are found.

### Electrical Properties of nMAG

**Fig. 4:**
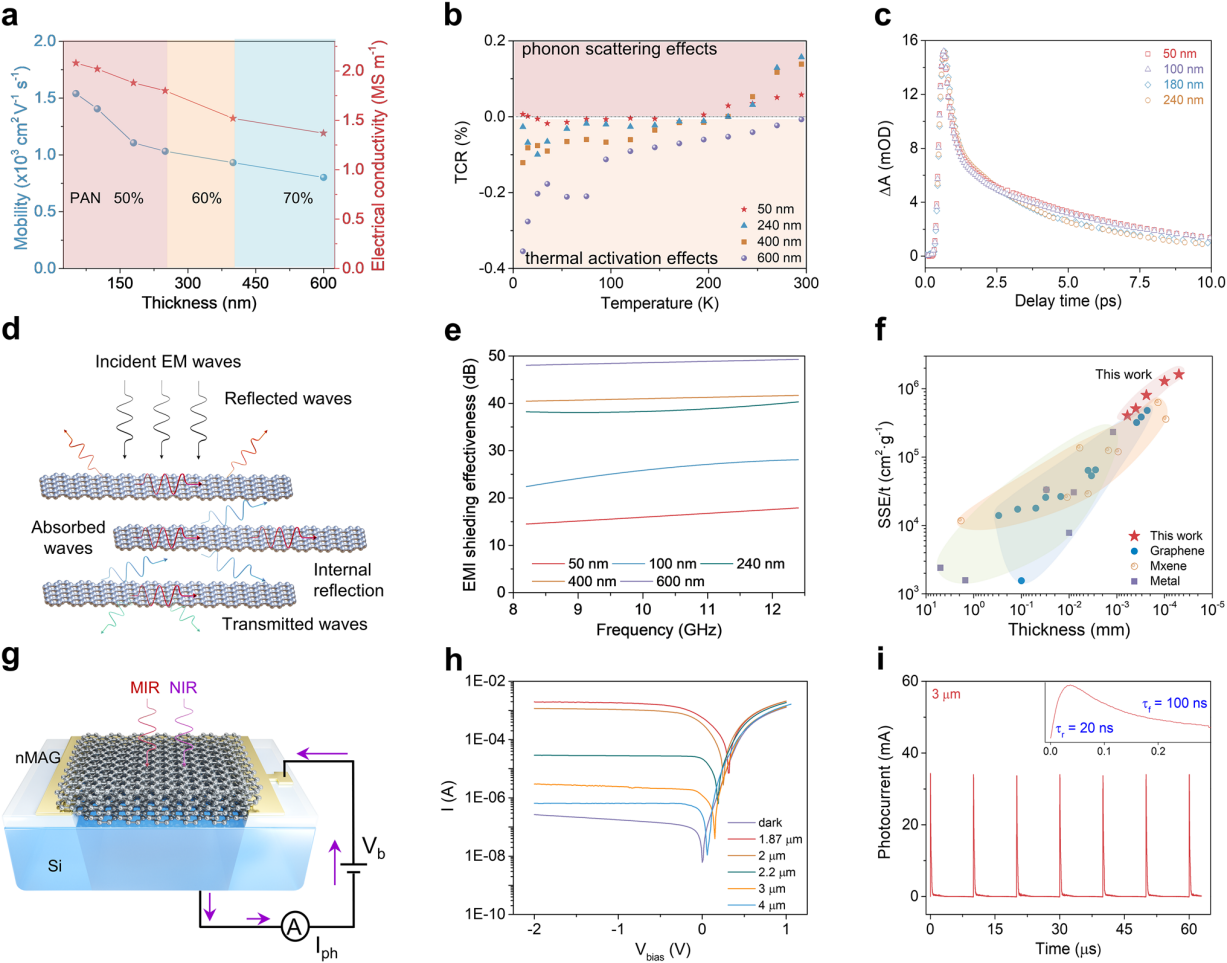
Electrical properties and applications of nMAGs. **a** Carrier mobility and electrical conductivity of nMAGs. **b** TCR of nMAGs with different thicknesses. **c** Transient absorption kinetics of nMAGs with different thicknesses (power density, 1 mW mm^−2^; pump wavelength, 532 nm; probe wavelength, 1.4 μm). **d** Schematic illustration of the electromagnetic shielding mechanism of nMAG. **e** EMI SE of nMAGs of different thicknesses of 50, 100, 240, 400, and 600 nm. **f** Comparison of SSE/t among different EMI materials (see Table S4 for details) in X-band versus thickness. **g** Schematic of the nMAG/Si Schottky diode. **h** *I-V* curves of nMAG/Si under laser illumination at different wavelengths (average power density 40 mW mm.^−2^). **i** Photocurrent of nMAG/Si in time domain under pulsed-laser (200 fs pulse width) illumination at 3 µm wavelength (τ_r_ = 20 ns is the rising time, which means the time of signal rising from lowest point to highest point)

The perfect graphite structure also endows nMAGs with excellent electrical properties. With the increase in thickness and PAN content, the carrier mobility/electrical conductivity is lowered from 1,540 cm^2^ V^−1^ s^−1^/2.04 MS m^−1^ to 802 cm^2^ V^−1^ s^−1^/1.37 MS m^−1^ (Fig. 4a and Table 3) due to the increased edge defects and stacking disorders (Fig. 3e), which is outperforming most of the graphene films with high-temperature thermal treatment. It should be noted that the redox reaction between GO and PAN during the pre-oxidation process occurs without adding chemical reducing agents. On the other hand, if HI is added as a reducing agent, cyclization is hindered, and the formation of a cross-linked 2D structure of PAN molecules cannot occur. This reduces the conductivity of the resulting nMAG (89 nm) from 2.01 MS m^−1^ (without HI reduction) to 1.75 MS m^−1^ (with HI reduction) (Fig. S17).

Due to its high crystallinity, an ultralow temperature coefficient of resistance (TCR) value of 0.04% at 10 K is observed in nMAG at 50 nm thickness (Fig. 4b). As the thickness increases from 50 to 600 nm, the TCR increases from 0.04% to 0.36% at 10 K. A continuous change in the conduction mechanism from thermal activation of carrier density to temperature-dependent phonon scattering effect accompanies this. At thicknesses smaller than 400 nm, due to the high purity and crystallinity (AB content > 92.7%), the conductivity is dominated by phonon scattering effects, and the thermal activation behavior is suppressed, which endows the nMAGs with a positive TCR (metallic behavior, Fig. 4b) in the temperature range of 200–300 K [28]. At temperatures below 200 K, thermal activation effects predominate. As the further temperature decreases, the electron hopping mechanism is modified from nearest-neighbor hopping to the Mott variable range hopping mode [29]. Both these modes lead to a negative TCR at low temperatures [29, 30]. In 600-nm-thick nMAGs, due to the relatively high level of disorder in interlayer stacking, the conductivity is predominantly due to the thermal excitation of carriers, because of which a negative TCR is observed at temperatures below 300 K. Furthermore, the absolute value of TCR is closely related to the mobility of the sample. In nMAGs, with thickness decreasing from 400 to 50 nm, there is a decrease in stacking disorder accompanied by increased mobility. Consequently, the activated conductivity shows a weak temperature dependence, or in other words, a lower absolute value of TCR [28]. Interestingly, for thickness < 240 nm, the TCR value stays near zero in the low-temperature range of 10 to 200 K. This behavior indicates that nMAGs have excellent potential for applications in carbon-based electronics working in deep space environments.

We measured the carrier transport behavior of nMAGs with thicknesses in the 50–240 nm range. As depicted in Figs. 4c and S18, the 50-nm-thick nMAG shows a lifetime of 4.7 ps, much longer than that of single-layer graphene (1 ps) [5]. The long carrier lifetime can be attributed to the high thickness and AB content of nMAGs. The high thickness provides a large cross section for carrier migration and reduces the specific surface area of graphene, significantly lowering the electron–phonon scattering at the surface. Besides, the high AB content endows nMAG with a strongly coupled electronic structure, weakening carrier scattering at disordered stacking interfaces in the out-of-plane direction. As the thickness increases from 50 to 240 nm, the increased disorder lowers the relaxation time to 4.3 ps. The long carrier cooling time promotes photo-thermionic (PTI) and carrier multiplication effects of nMAG [31], both of which are expected to be advantageous for next-generation optoelectronic devices operating in mid-infrared to X-ray regions [32].

### Interaction of nMAG with Electromagnetic Waves

The highly crystalline nMAGs interact strongly with electromagnetic waves from the X-band to the infrared region. The high electrical conductivity of nMAGs makes them particularly interesting for application in EMI shielding (Fig. 4d). As shown in Fig. 4e, a 100-nm-thick nMAG offers an EMI total shielding effectiveness (SE_T_) of 25.84 dB in the X-band frequency range (8.2 to 12.4 GHz), which is greater than the commercial EMI shielding requirement (> 20 dB) [14]. A vast majority of the incident EM waves are reflected because of the abundant free electrons and electric dipoles with a power coefficient of reflection (*R*) of 0.90 (Fig. S20a) [15]. The residual EM waves enter the interior of the nMAG and interact with the induced current generated by the nanofilm, consuming electromagnetic energy and generating heat (Fig. 4d). With the increase in thickness, the decreased conductivity of nMAG lowers the power coefficient of reflection (*R*) and the reflection loss (*SE*_R_) slightly [33]. Due to this effect, the rate of *SE*_T_ increase with thickness is reduced, especially in the thickness range of 240–600 nm (Fig. S20b). Besides, the consumption of electromagnetic energy increases due to the combined influence of conductivity and thickness, which is confirmed by the improved absorption (*A*) and absorption loss (*SE*_A_) power coefficients. When the thickness is increased to 600 nm, the EMI *SE*_T_ increases to 48.57 dB. This high *SE*_T_ could be promising for applications in advanced intelligent electronics.

Miniaturized electronic devices, such as portable and wearable electronics, require EMI shielding materials with a lightweight, minimal thickness (*t*) and flexibility, along with high EMI *SE*_T_. Therefore, to evaluate the performance of nMAGs, we adopted an absolute EMI shielding effectiveness value defined by dividing EMI *SE*_T_ by the density (*ρ*) and thickness (*t*) as a standard reference [34]. As shown in Fig. 4f, the ultrathin 50-nm-thick nMAG shows an outstanding absolute effectiveness of shielding (*SSE/t*) of 1,619,000 dB cm^2^ g^−1^ in the X-band, significantly outperforming MXene- and carbon-based materials, so far reported in the 50–600 nm thickness range (Table S4) [9, 14, 15, 35–54].

In the infrared region, the 50-nm-thick nMAG shows a strong absorption of 44.6–46.1% in the wavelength range of 2–10 μm (Fig. S21). We integrated the 50-nm-thick nMAG with silicon into a Schottky heterojunction with van der Waals contact **(**Fig. 4g) [5]. The HR-TEM image of the nMAG/Si cross section confirms the highly ordered structure of nMAG and indicates an atomic-scale interfacial contact arising from the high surface energy of the 50-nm-thick nMAG (Fig. S22). Compared to single-layer graphene, the bulk structure with AB stacking geometry provides a larger cross section for light absorption and carrier multiplication, which together endow nMAG with a strong PTI emission effect [55]. The low-energy photo-excited carriers in nMAG can thermalize by Auger scattering into a Fermi–Dirac distribution, and a fraction of hot electrons will be excited to energy higher than the Schottky barrier height of nMAG/Si, thus extending the response wavelength of the detector and overcoming the limitation of Si bandgap (1.2 eV) [56]. Based on its strong PTI effect, the nMAG/Si device shows broadband photoresponse in the wavelength range of 1.87–4.0 μm (Fig. 4h), a much broader range than single-layer graphene/Si diodes (< 1.5 μm) with the same device structure and working conditions. The assembled Schottky diode shows decreased responsivity (4.9 × 10^–2^ to 9.7 × 10^–6^ A W^−1^) and detectivity (2.7 × 10^10^ to 5.3 × 10^6^ Jones) from 1.87 to 4.0 µm. Furthermore, we explored ultrafast optoelectronic dynamics in nMAG/Si using femtosecond (pulse width, Δ*t* = 200 fs) photoexcitation in MIR. A response time of *τ*_r_ ~ 20 ns (Fig. 4i) is observed at the wavelength of 3 μm, determined by device structure (window size and non-depleted region width) and resistance in external circuits and [57]. These performances of nMAG/Si can lead to their application in broadband and ultrafast MIR active imaging devices working at room temperature.

### Thermal Conducting Performance of Assembled 10 μm-thick mMAG Film

**Fig. 5:**
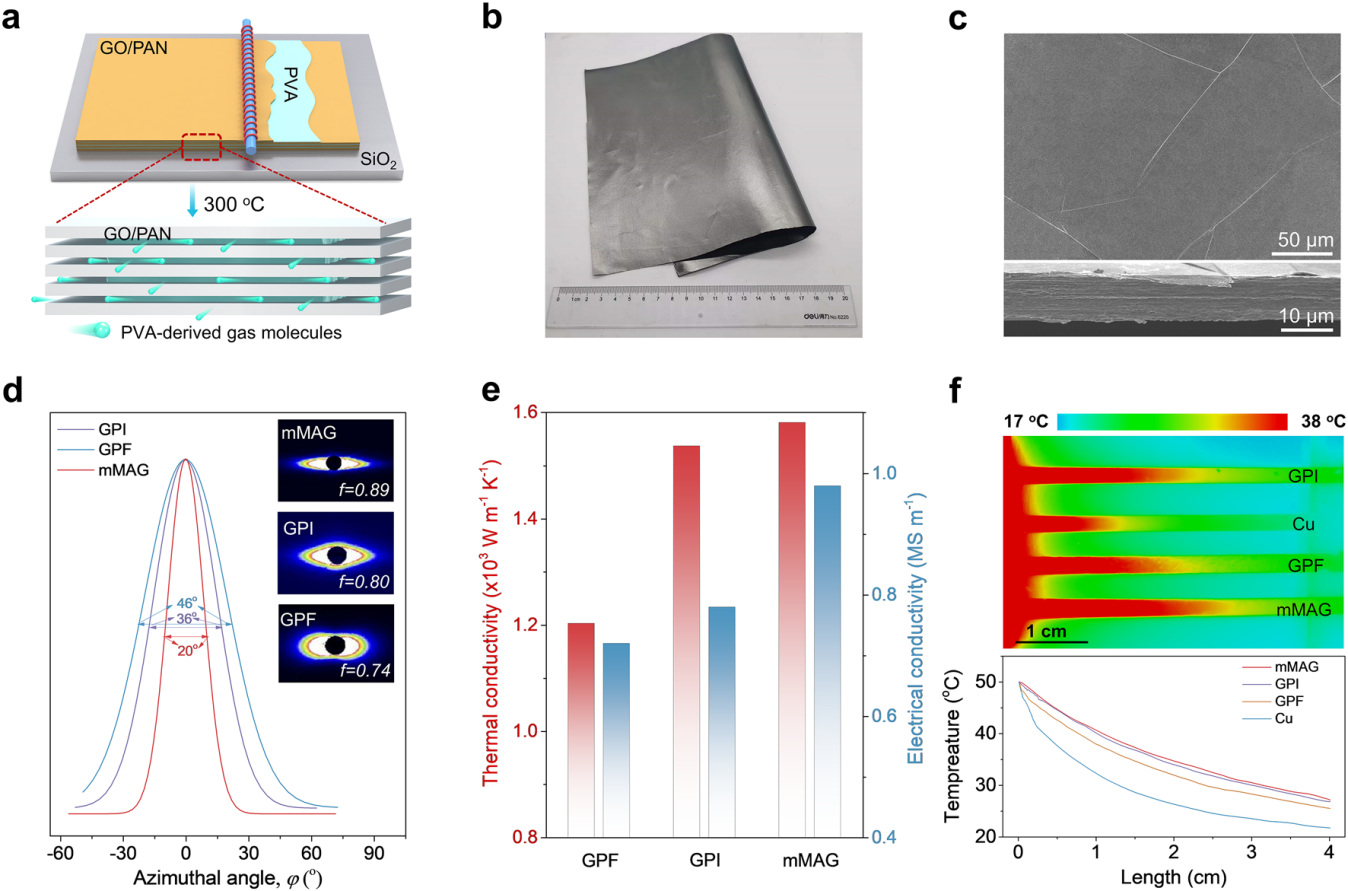
Thermal properties of 10-μm-thick mMAG assembled from 200-nm-thick nMAGs. **a** Schematic diagram of the layer-by-layer assembly of GO/PAN film (400 nm per layer) and PVA (5–20 nm per layer) to form a 10-μm-thick mMAG after 3,000 °C heat treatment (Up) and a schematic diagram of PVA decomposition during the pre-oxidation process (Down); yellow and gray films are PAN/GO films before and after pre-oxidation, and the green ones are PVA. **b** Optical image of mMAG. **c** SEM images of mMAG surface and cross-section, showing the ultralow density of microfolds. **d** Azimuthal angle (*φ*) plots of scattering patterns by SAXS and the corresponding 2D SAXS patterns (insets) for mMAG, GPI, and GPF films. **e** Thermal and electrical conductivities of mMAG, GPI, and GPF films. **f** Infrared thermal images and the corresponding temperature profiles of 10-μm-thick GPI, Cu, GPF, and mMAG with one side tightly attached to a thermostatic heater

nMAGs can be used as building blocks to construct high-performance thermal management materials with sufficient heat flux. However, in nMAGs with thickness > 600 nm, the atomic-scale channels present do not efficiently allow gases to escape during annealing, resulting in a lowered performance. To obtain graphene films with micro-scale thickness and high thermal conductivity, we assembled 400-nm-thick (230 nm after 3,000 °C) GO/PAN nanofilms and 5–20-nm-thick polyvinyl acetate (PVA) nanolayers layer by layer to construct films with hierarchical channels (Fig. 5a). During pre-oxidation of the assembled GO/PAN-PVA film (180–300 °C), the decomposition of PVA at 240 °C is accompanied by the release of gaseous molecules (CO_2_, H_2_O, etc.), which delaminates the GO/PAN layers and generates flat gas channels. Note that almost no PVA residue was found after 1,000 °C treatment [58]. As a result, the layered thick film does not have micro-gasbag structures (Fig. S23a), which means that no wrinkles will be introduced during the densification of the film. The rich channels facilitate gas to escape in the GO/PAN layer during the subsequent annealing process. After heat treatment at 3000 °C and pressing at 300 MPa, large-area micron-scale-thick macro-assembled graphene film (mMAG) with 10 μm thickness was obtained (Fig. 5b). As shown in Figs. 5c and S23b**,** the hierarchical channels to efficiently escape gases suppress the formation of surface wrinkles to a large extent. The microfold density of mMAG (11.4 mm mm^−2^) is about 9.3% of that of conventional PAN/GO-based graphite films (GPF) without PVA and 21.3% of that of commercial artificial PI-based graphite film (GPI) with the same thickness (Fig. S24).

SAXS patterns and the corresponding azimuthal scanning curves were used to reveal the stacking nature of graphene sheets. As shown in Fig. 5d, mMAG exhibits a higher value of Herman’s orientation factor (0.89) and a narrower azimuthal distribution (FWHM, 20°) than GPI (0.80, 36°) and GPF (0.74, 46°) [59]. The near absence of micro-wrinkles allows for a higher orientation degree of graphene sheets in mMAG, reducing electron and phonon transport path lengths. Thus, mMAG shows an in-plane thermal conductivity of 1,581 W m^−1^ K^−1^ and electrical conductivity of 0.98 MS m^−1^, outperforming both GPI (1,537 W m^−1^ K^−1^, 0.78 MS m^−1^) and GPF (1,203 W m^−1^ K^−1^, 0.72 MS m^−1^) with the same thickness (Fig. 5e). Besides, the 10-μm-thick GPF derived from the HI-reduced GO/PAN film shows a low in-plane thermal conductivity (981 W m^−1^ K^−1^) and electrical conductivity (0.53 MS m^−1^), which further confirms the inhibitory effect of HI on graphitization of PAN molecules. Infrared images confirm the high heat-transfer speed of our mMAG, which is faster than those of GPF and copper foil (Cu) and comparable to that of GPI (Fig. 5f). We note here that our fabrication process for mMAG does not require expensive biaxial stretching equipment for the orientation of the polymer and is, therefore, more amenable to industrial production than GPF.

## Conclusions

Using a simple ‘substrate replacement’ strategy, we prepared large-area, self-standing, and highly crystalline graphene nanofilms in a broad thickness range (50–600 nm). The successful preparation of nMAG films is attributed to the introduction of PAN, which promotes interfacial separation with substrate and allows gas escape before complete transformation to intact graphene lattice. Due to close-packed and highly crystalline structure, nMAGs exhibit excellent conductivity and carrier mobility of (1,540 cm^2^ V^−1^ s^−1^/2.04 MS m^−1^ to 802 cm^2^ V^−1^ s^−1^/1.37 MS m^−1^) with static TCR, a solid light–matter interaction (photoelectric conversion capability in the mid-infrared and EMI shielding effectiveness in X-band), superior flexibility and high thermal conductivity (1,581 W m^−1^ K^−1^) when assembled into 10-µm-thick graphene films. Our synthesis strategy is suitable for a wide range of multifunctional bulk nanofilms and is expected to enable their practical applications as electronic and optoelectronic platforms in the future.

### Supplementary Information

Below is the link to the electronic supplementary material.

Supplementary file1 (DOCX 35197 KB)
