# Supplementary material for: Flexible Large-Area Graphene Films of 50–600 nm Thickness with High Carrier Mobility
Source: Nanomicro Lett. 2023 Mar 3;15:61. doi: 10.1007/s40820-023-01032-6 (PMC9984600; doi:10.1007/s40820-023-01032-6)
Supplement: Supplementary file 1 — Supplementary file1 (DOCX 35197 KB) [file 40820_2023_1032_MOESM1_ESM.docx]

*Supporting Information*

**Flexible large-area graphene films of 50-600 nm-thickness with high carrier mobility**

*Shiyu Luo^1^, Li Peng^1,2^*, Yangsu Xie^3^, Xiaoxue Cao^1^, Xiao Wang^4^, Xiaoting Liu^1^, Wenzhang Fang^1^, Tingting Chen^3^, Zhanpo Han^2^, Peidong Fan^5^, Haiyan Sun^5^, Ying Shen^1^, Fan Guo^1^, Yuxing Xia^1^, Kaiwen Li^1^, Xin Ming^1^, Chao Gao^1^**

^1^MOE Key Laboratory of Macromolecular Synthesis and Functionalization, Department of Polymer Science and Engineering, Zhejiang University, Hangzhou 310027, China.

^2^ZJU-Hangzhou Global Scientific and Technological Innovation Center, School of Micro-nanoelectronics, Zhejiang University, Hangzhou 310027, China.

^3^College of Chemistry and Environmental Engineering, Shenzhen University, Shenzhen, Guangdong 518055, China.

^4^Shenzhen Key Laboratory of Nanobiomechanics, Shenzhen Institute of Advanced Technology, Chinese Academy of Sciences, Shenzhen 518055, China.

^5^Hangzhou Gaoxi Technol Co Ltd, Hangzhou 311113, China.

* Corresponding author. E-mail: Li Peng (l-peng@zju.edu.cn)

Chao Gao. (chaogao@zju.edu.cn)


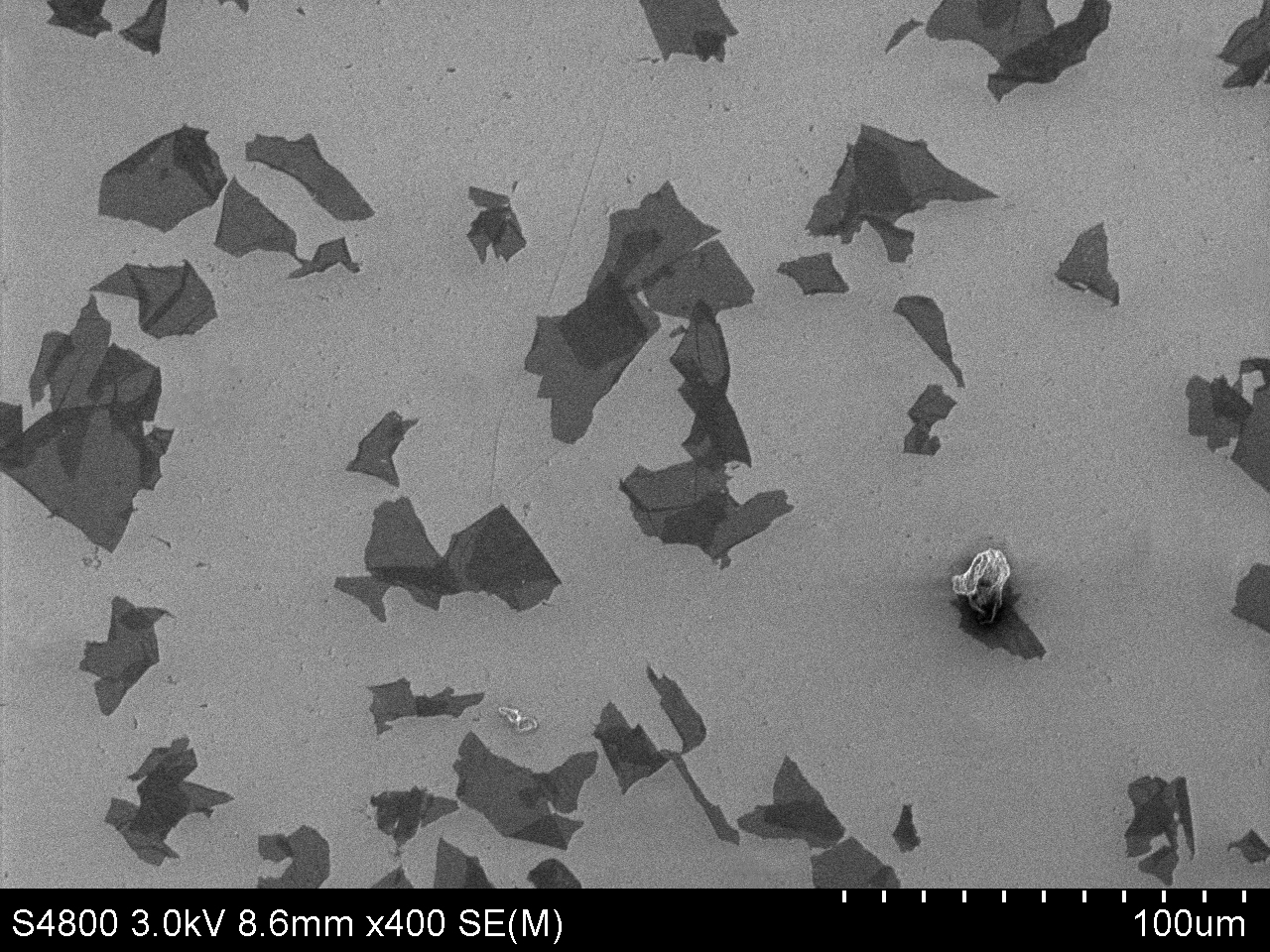

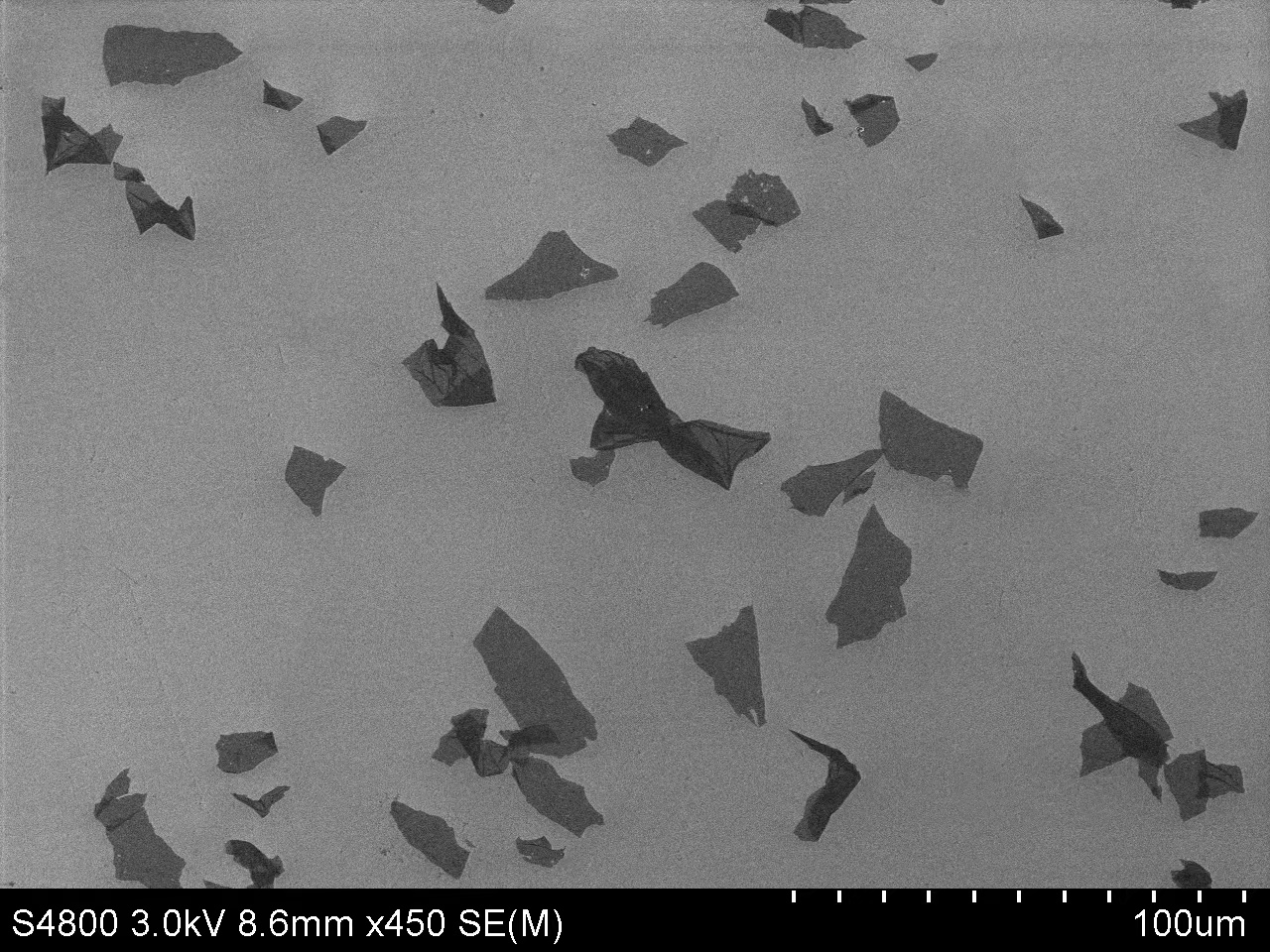

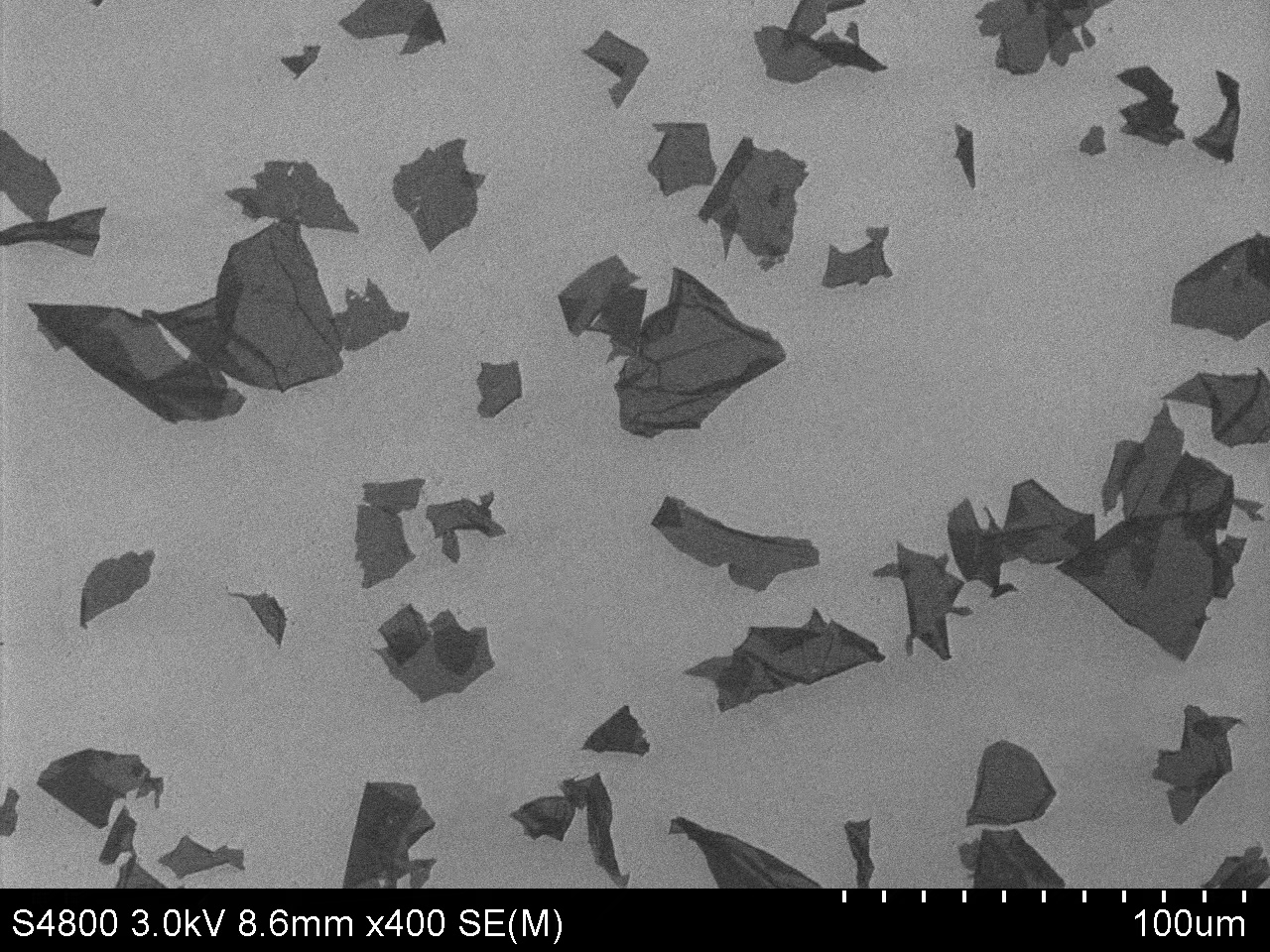


50 μm

50 μm

50 μm

**Figure S1.** SEM images and lateral size distribution of GO sheets.


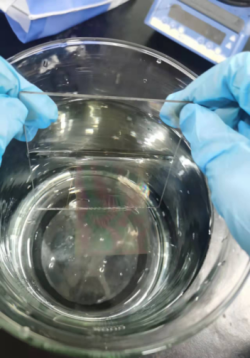

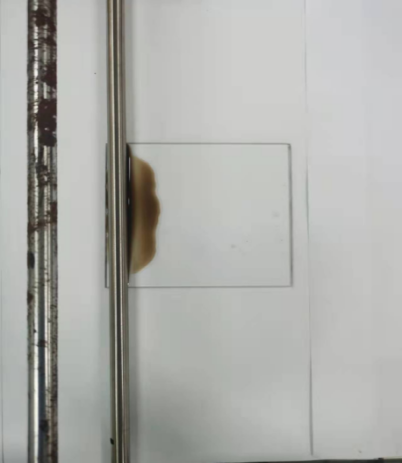


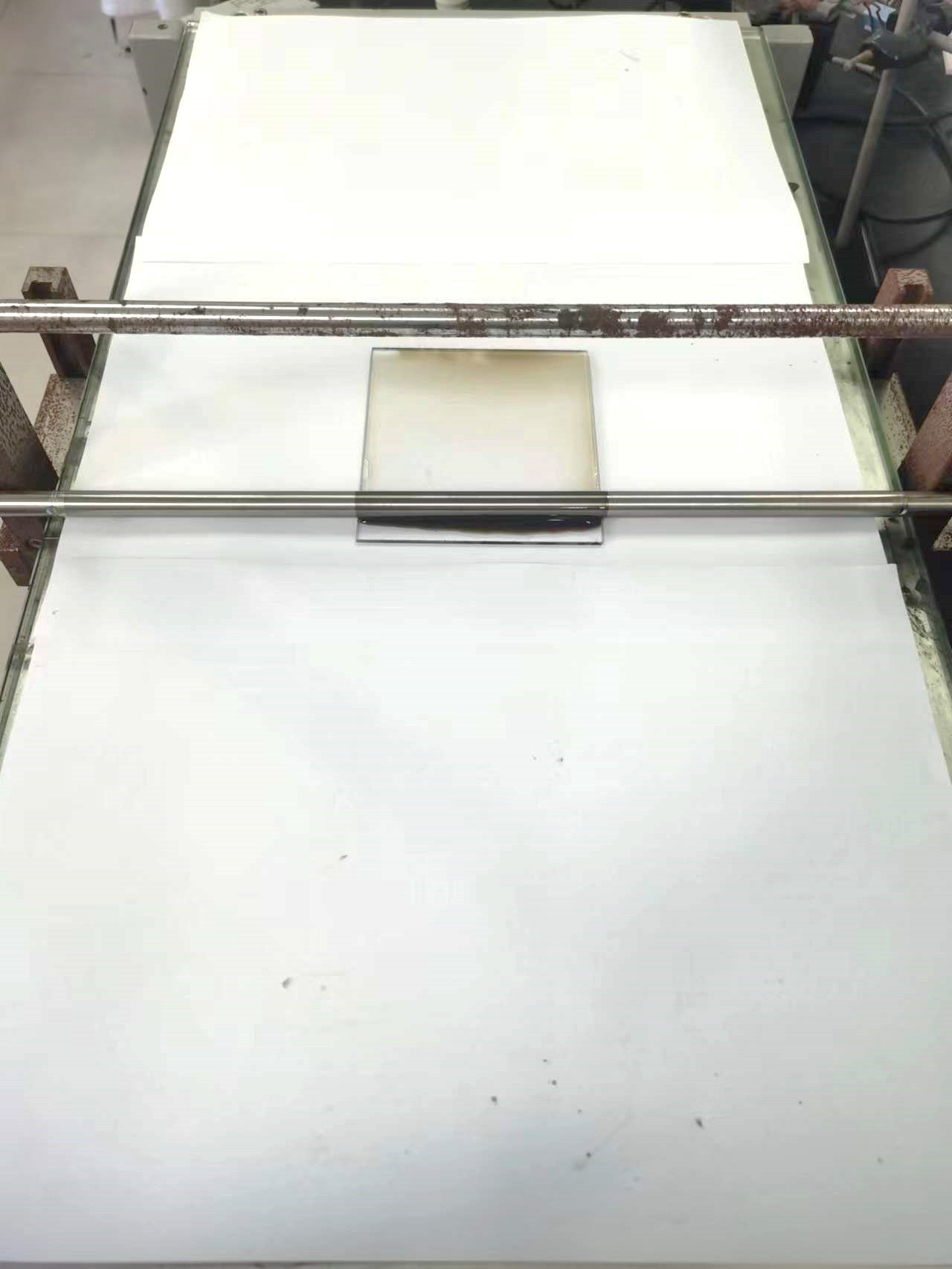


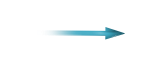


peel off


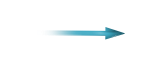


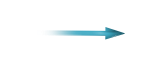


exchange solvent


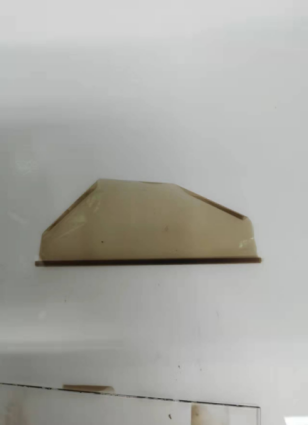

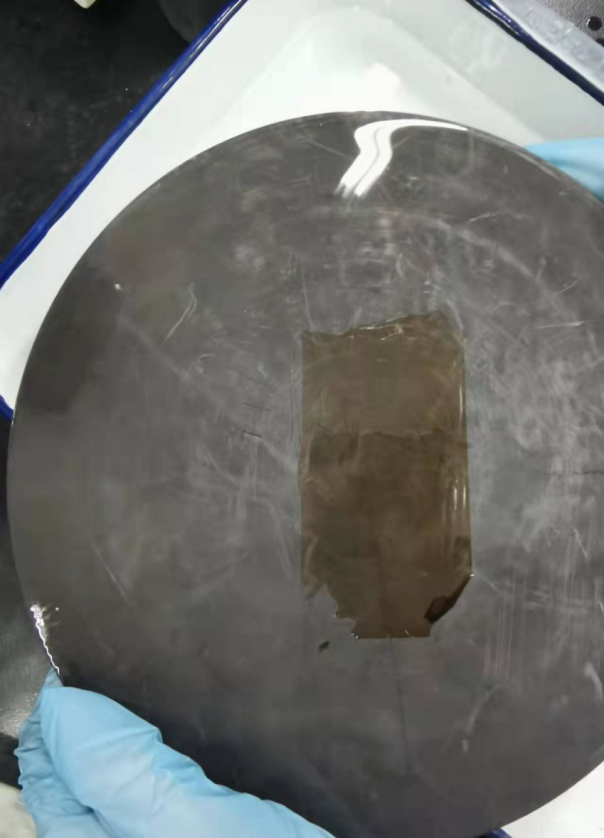

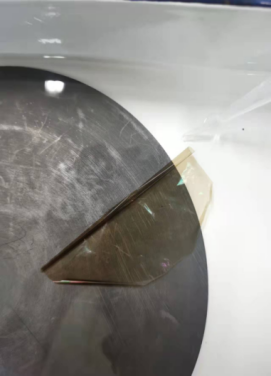


dry


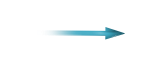

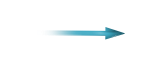


graphite plate

**Figure S2.** The preparation process of free-standing GO/PAN film.





**a b c**

quartz plate


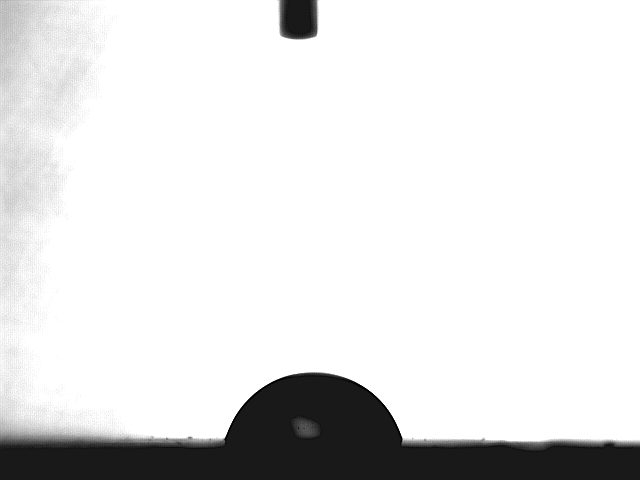


69^o^


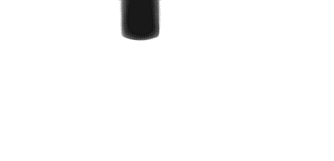


GO/PAN film

500 μm

**Figure S3.** (a) SEM image of GO/PAN film exfoliated by water. (b) The water contact angle of the quartz plate. (c) XRD patterns of GO/PAN films with different PAN contents before and after swelling by water (unit: nm).

**a b c**


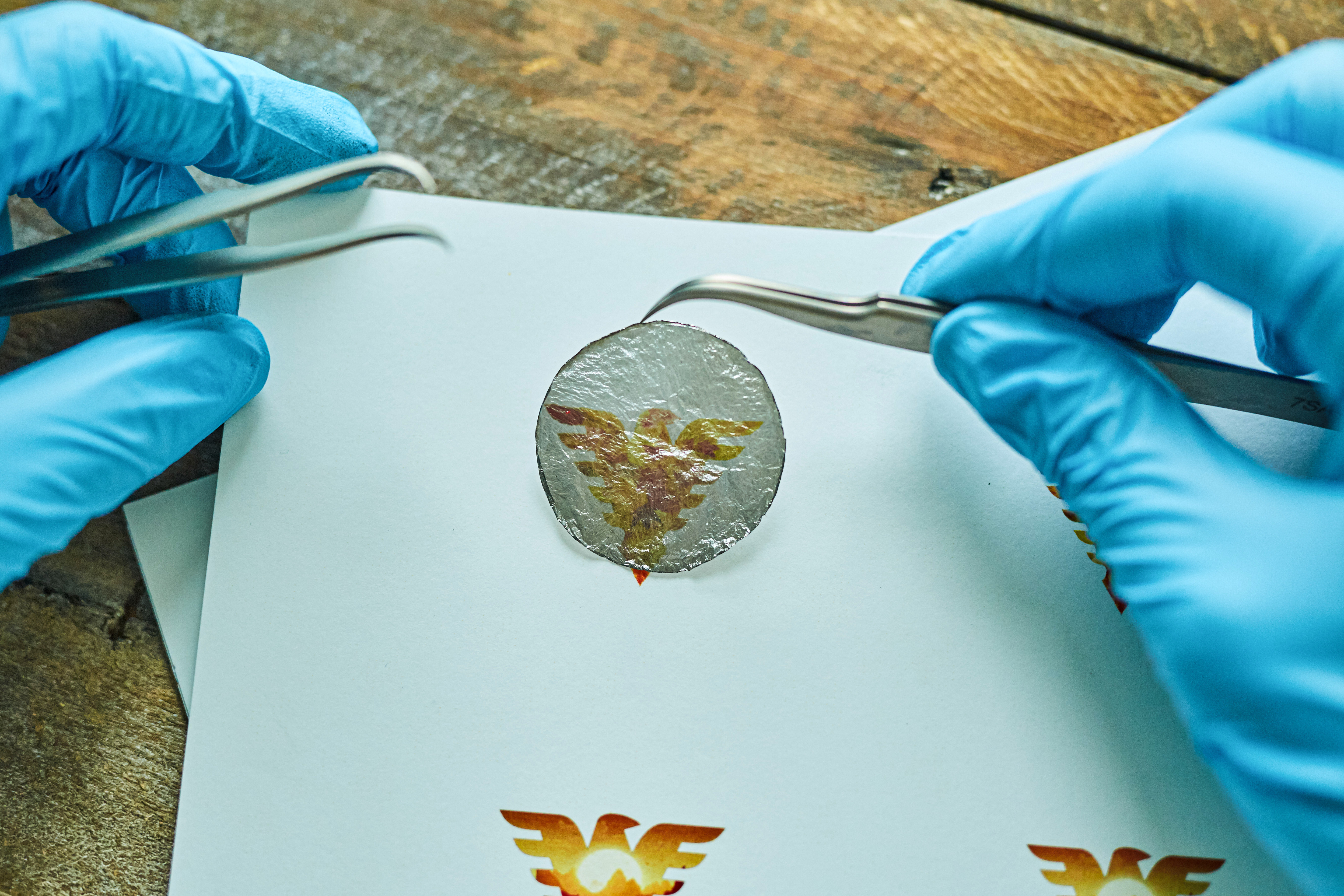


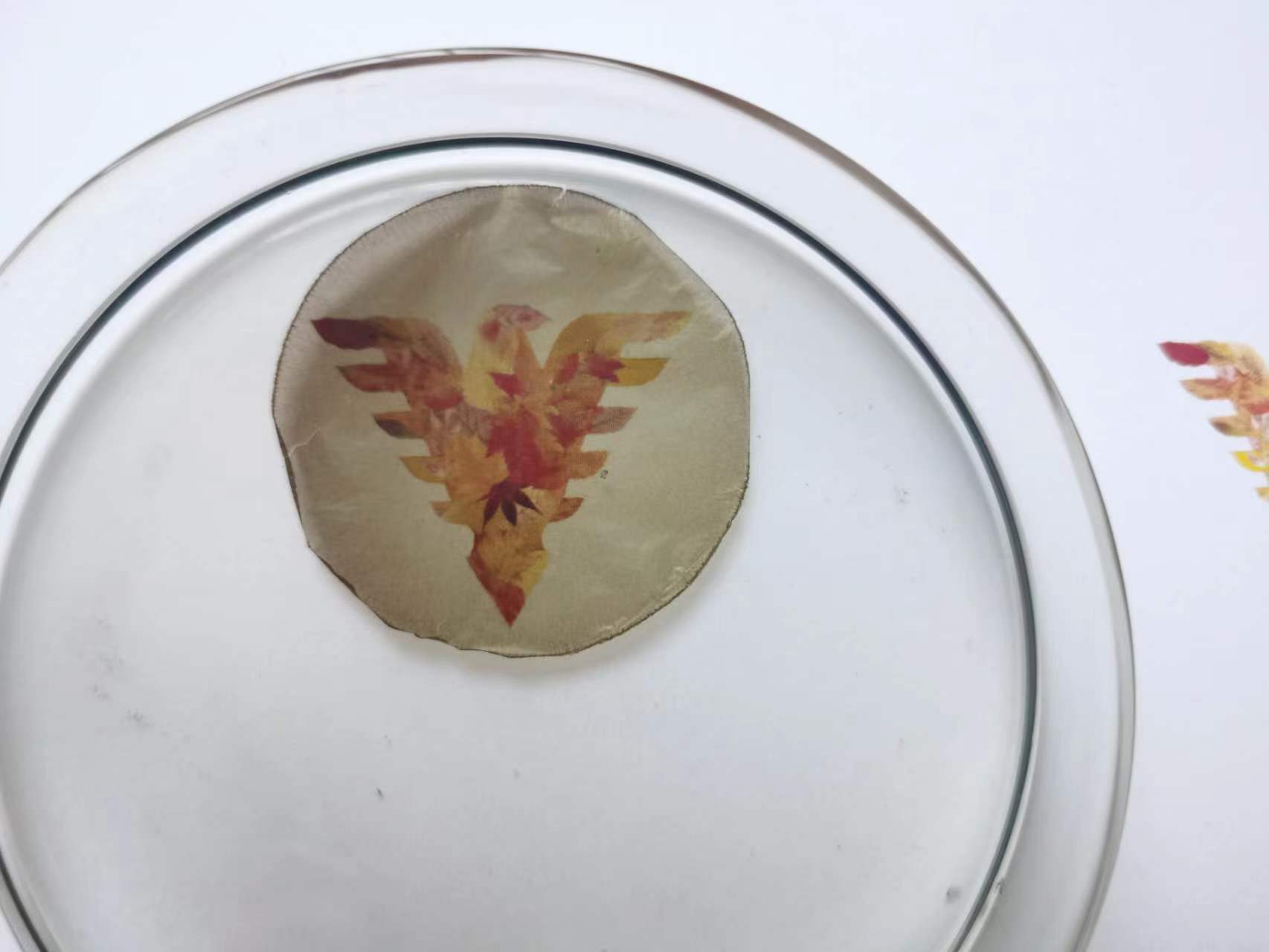

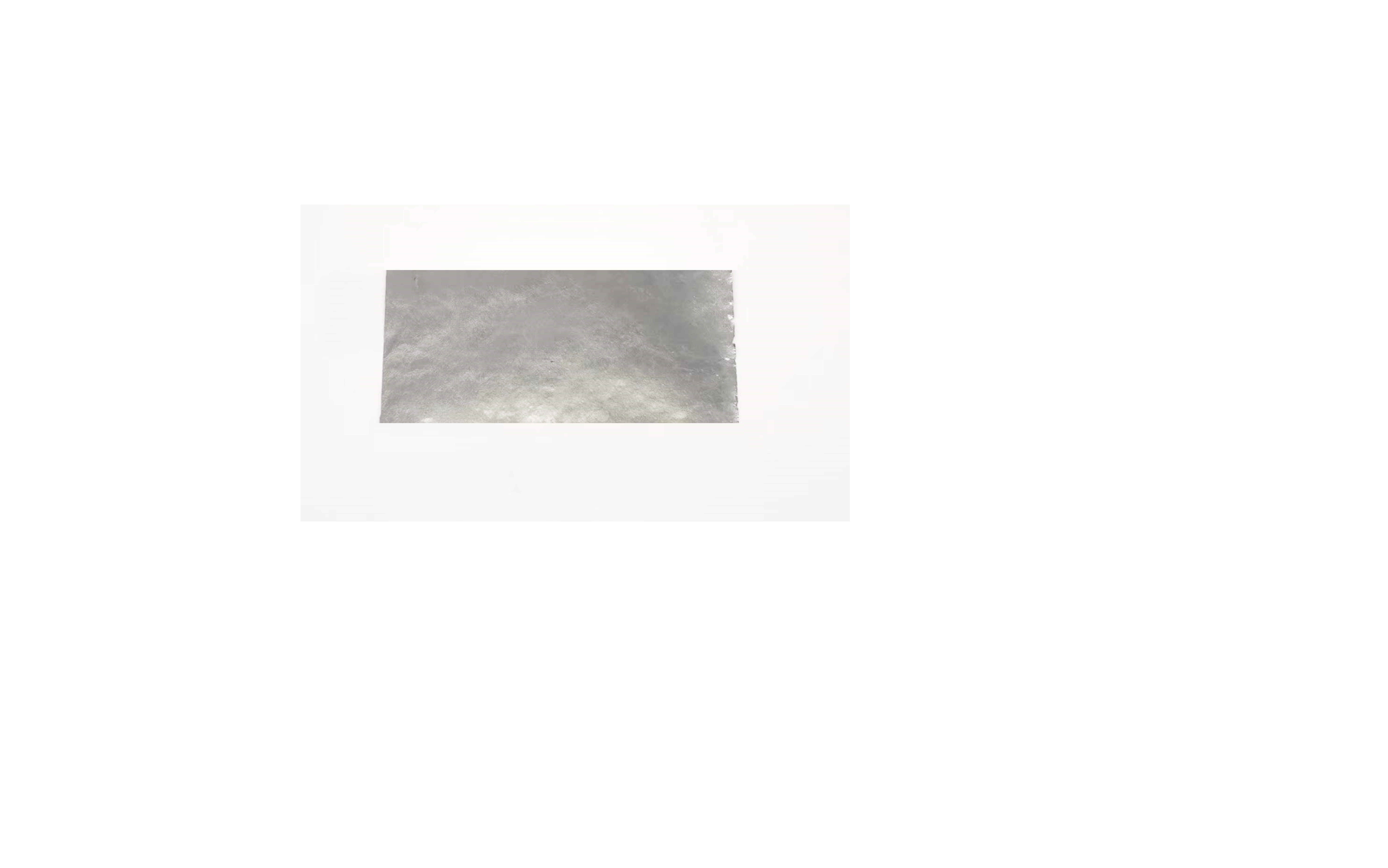


**Figure S4.** 50 nm-thick nMAG with a round shape before (a) and after (b) 3000 ^o^C heat treatment. (c) 600 nm-thick nMAG with a rectangle shape after 3000 ^o^C heat treatment.


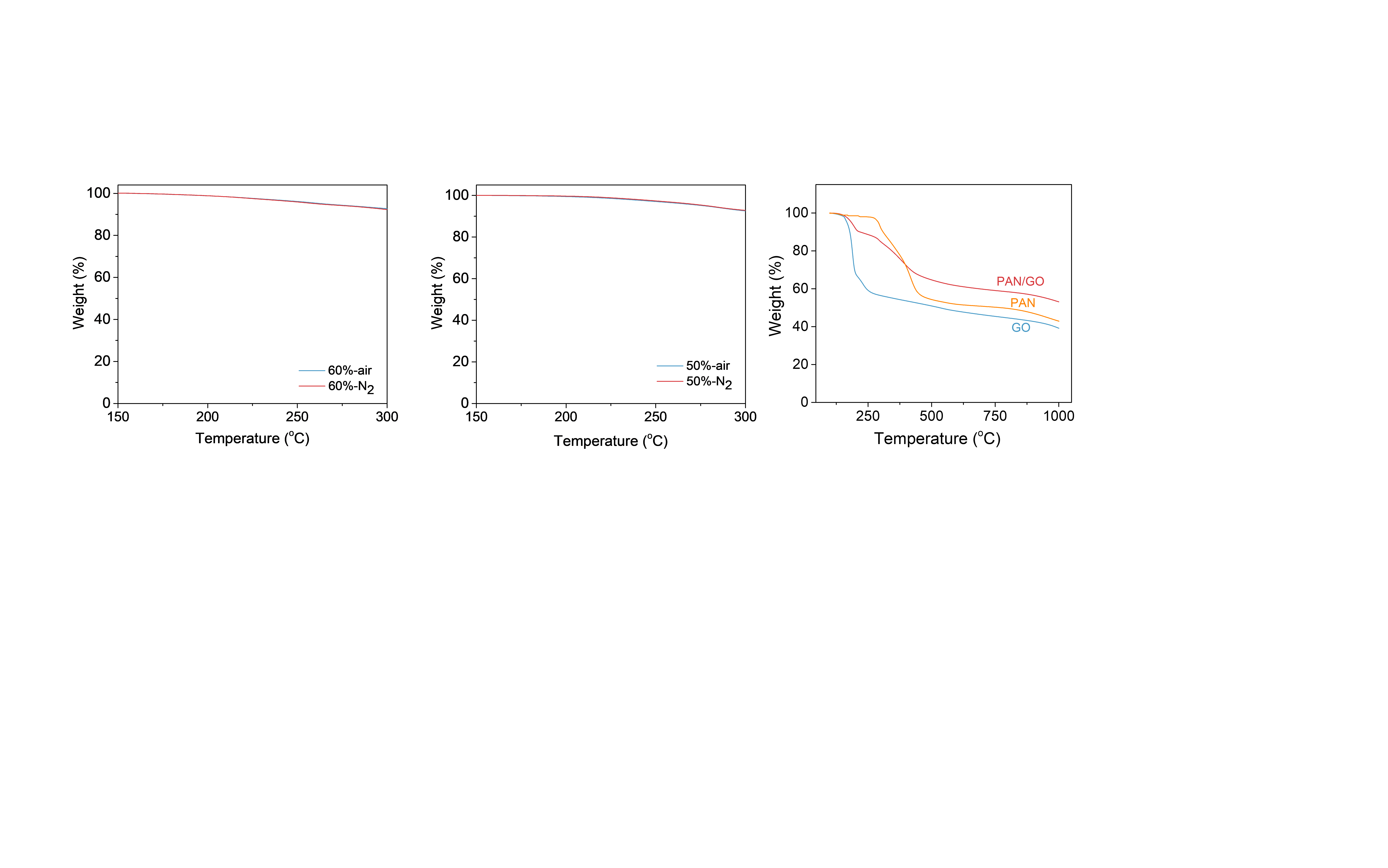


**a b**

**Figure S5.** (a) TGA plot of GO/PAN (PAN, 60%) in N_2_ and air atmosphere at 150–300 ^o^C. (b) The TGA plot of GO/PAN (PAN, 50%) in N_2_ and air atmospheres at 150–300 ^o^C.

**a**


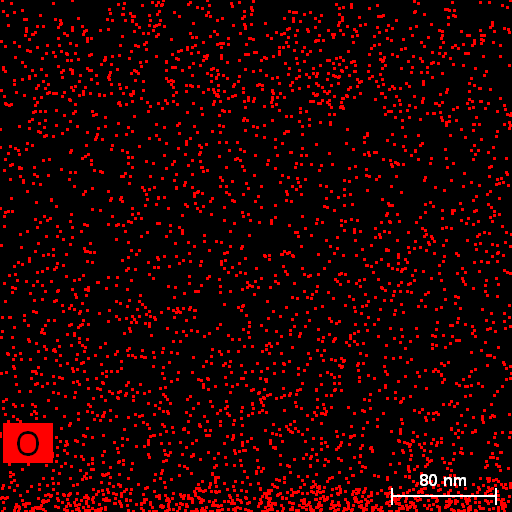

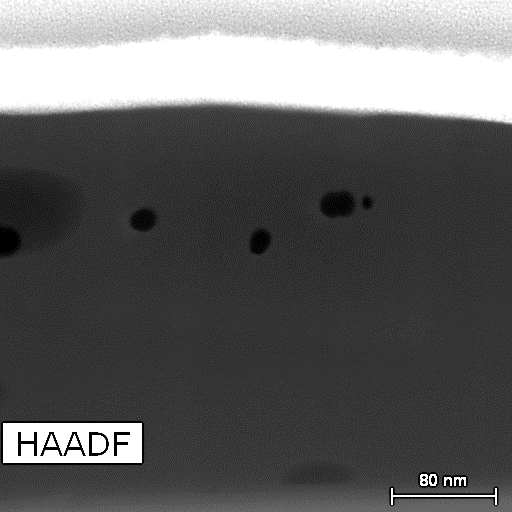


**PAN**

core

skin

**b**


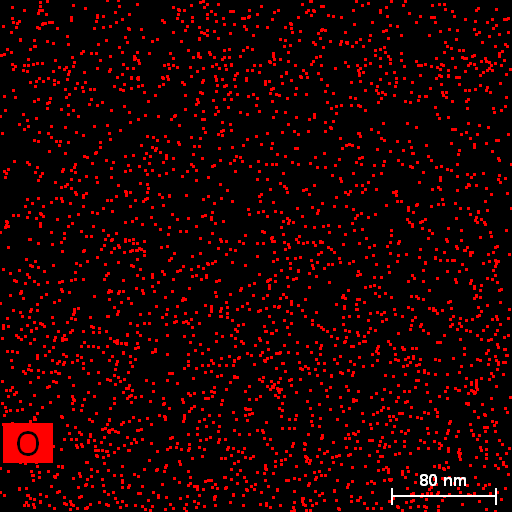

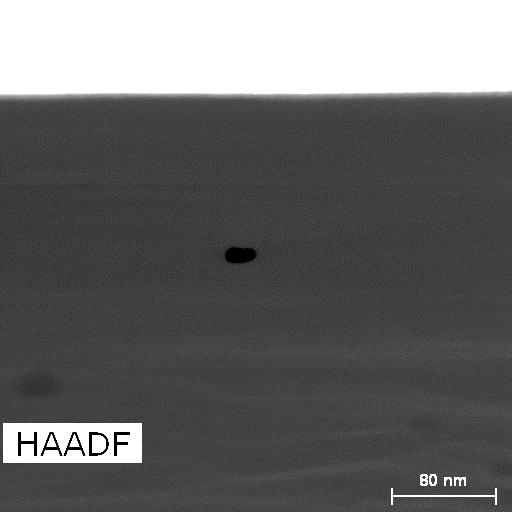


core

**GO/PAN (PAN, 50%)**

skin

**Figure S6.** Cross-sectional STEM image of (a) PAN and (b) GO/PAN (PAN, 50%) after pre-oxidation at 270 ^o^C and the corresponding oxygen EDS mapping image, indicating a “skin-core structure” in PAN.

**a b**

**c d d**

**Figure S7**. (a) XPS surveys of the nMAG (PAN, 50%) with different heat treatments. (b-d) XPS C1s spectra of the nMAG (PAN, 50%) with different heat treatments, respectively. The circles are experimental data, and the red line is the fitted curve.

**Figure S8**. The FTIR spectra of nMAG (PAN, 50%) with different heat treatments.

**a b**

**Figure S9.** XRD patterns of GO/PAN films before (a) and after (b) pre-oxidation.

**Figure S10.** (O+N)/C ratios of rGO and nMAG (PAN, 50%) elaborated by XPS.


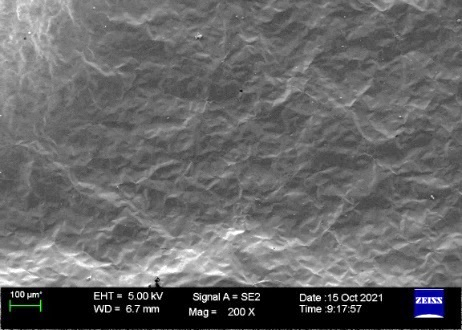


**a b c**


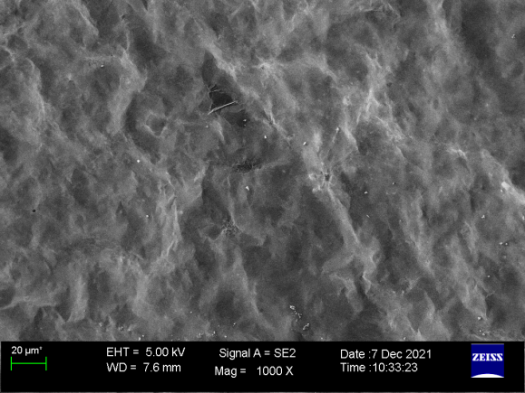

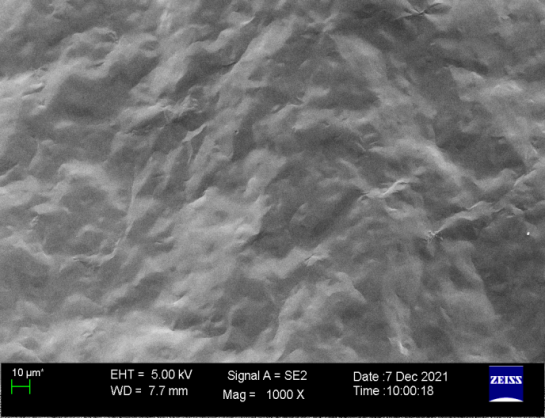


40 μm

40 μm

40 μm


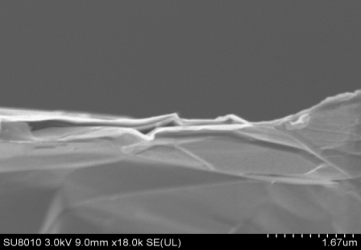

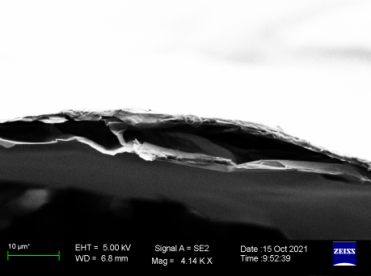
**
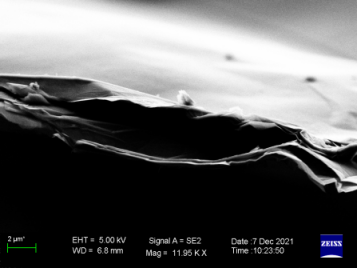
**

10 μm

1 μm

1 μm

**Figure S11.** (a) Surface and cross-sectional SEM images of 250 nm-thick nMAG (PAN content, 50%). (b) Surface and cross-sectional SEM images of 450 nm-thick nMAG (PAN content, 60%). (c) Surface and cross-sectional SEM images of 650 nm-thick nMAG (PAN content, 70%).

**a b**


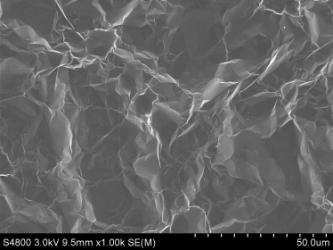



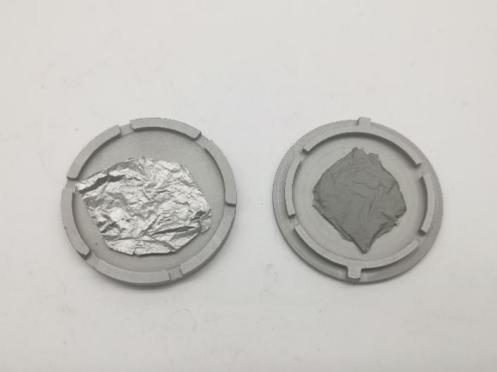


2 cm

20 μm

1 μm

**Figure S12.** (a) Surface and cross-sectional SEM images of 50 nm-thick GO-based graphene nanofilm obtained by vacuum-filtration. (b) Digital photo of nMAG (PAN, 50%, left) and GO-based graphene nanofilm (right) with the same thickness of 200 nm after heat treatment at 3000 ^o^C.

**a b**


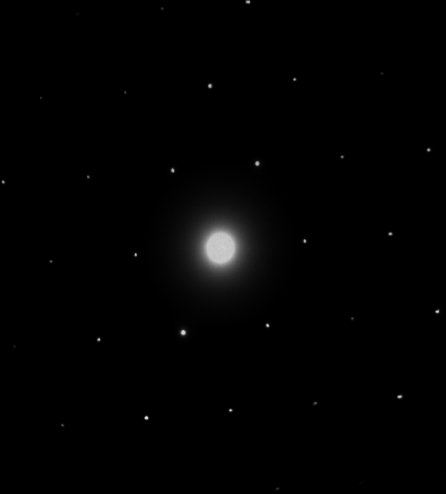

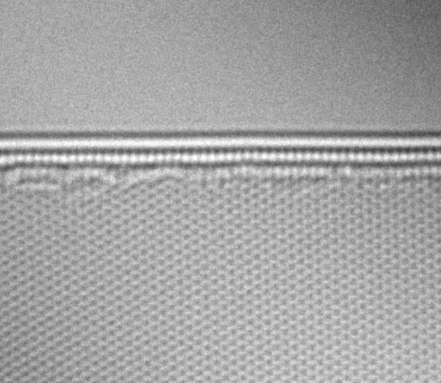

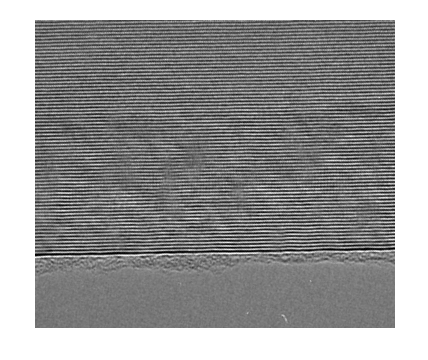

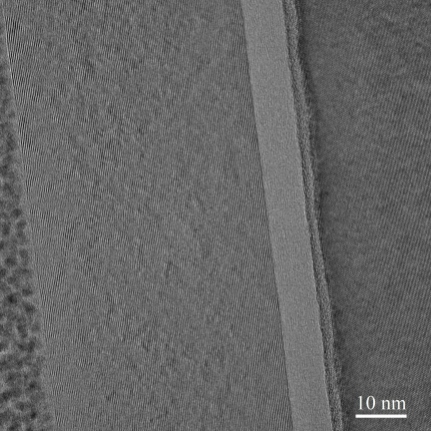


0.335 nm

2 nm

4 nm

15 nm

**c**


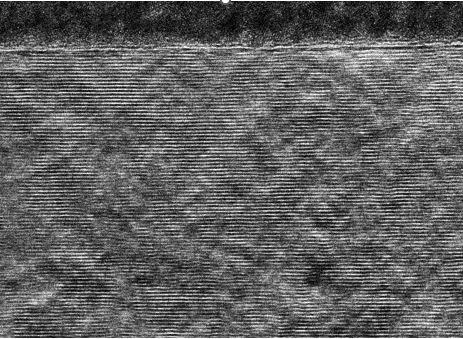

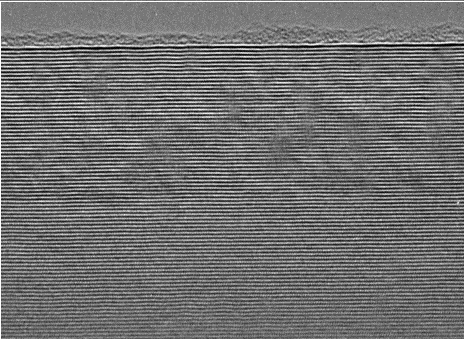


10 nm

10 nm

**Figure S13.** (a) Cross-sectional TEM image of 50 nm-thick nMAG with a graphite-like *d*-spacing. (b) Surface High-Resolution TEM image of graphene sheets in 50 nm-thick nMAG with perfect in-plane lattice and the corresponding selected area electron diffraction pattern (inset), which indicates an AB stacking geometry. (c) Cross-sectional TEM images of nMAG with parallel graphene lattices.

(006)


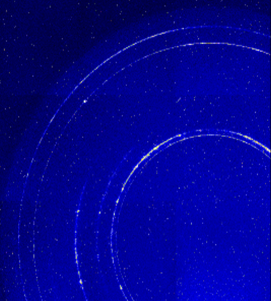


(004)

444)

(101)

444)

(100)

444)

(103)

(102)

(110)

(112)

**Figure S14.** 2D synchrotron WAXS patterns of 50-thick nMAG treated under 3000 ^o^C.

**a b**

**Figure S15.** (a) Raman spectra of GO, PAN, GO/PAN (PAN, 50%), and 270 ^o^C-treated GO/PAN (PAN, 50%) films. (b) XRD patterns of GO, PAN, and 3000 ^o^C-treated nMAG.

**a b**

**
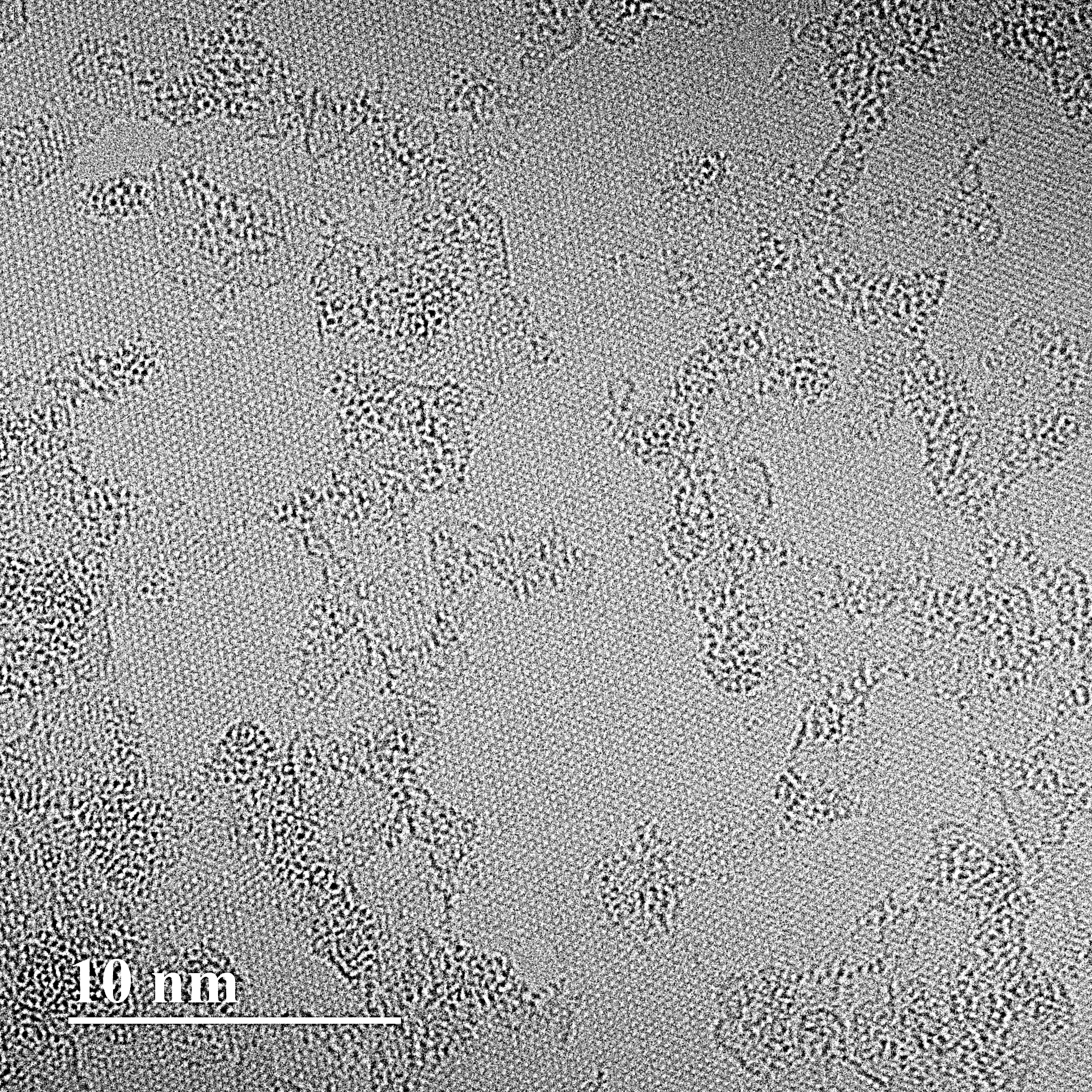

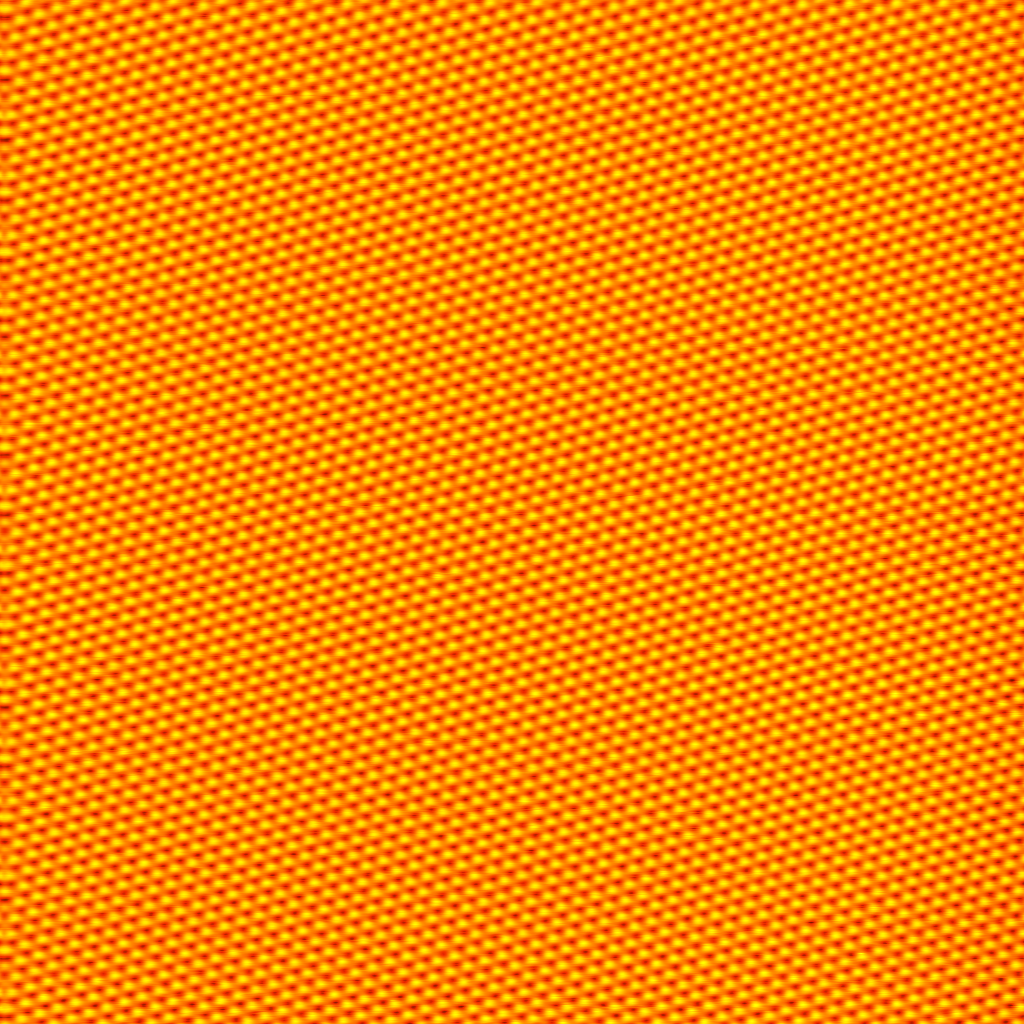
**

2 nm

4 nm

**Figure S16.** (a) HR-TEM image of GO. (b) STM images of 50 nm-thick nMAG surface.

**Figure S17.** Electronic conductivity of HI-reduced GO/PAN films of different thicknesses (PAN, 50%) heat-treated at 3000 ^o^C.

**Figure S18.** 2D transient absorption map of nMAG/Si as a function of delay time and incident photon energy.

**Figure S19.** EMI SE_T_ of PI substrate in the frequency range of 8.2-12.4 GHz.

**a b**

.

**Figure S20.** (a) Calculated EMI shielding coefficients of nMAGs in the X-band. (b) SE_R_, SE_A_, and SE_T_ values of nMAGs with different thicknesses of 50 nm, 100 nm, 240 nm, 400 nm, and 600 nm in the X-band.

**a b**

**Figure S21.** Fourier transform infrared (FTIR) absorption spectrum (a) and reflection spectrum (b) of 50 nm-thick nMAG.


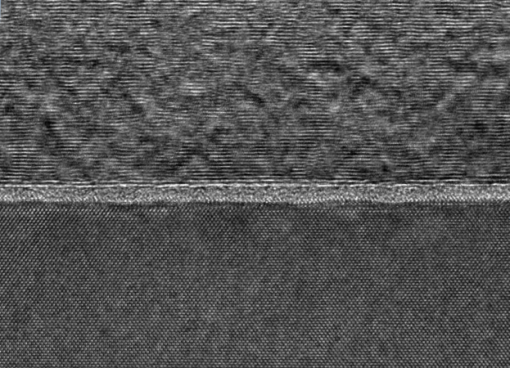


2 nm

**Figure S22.** High-Resolution TEM image of nMAG/Si cross-section.


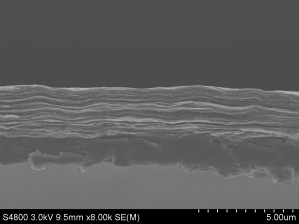


**a**

**b**
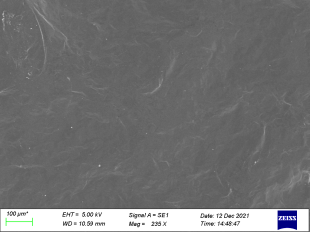


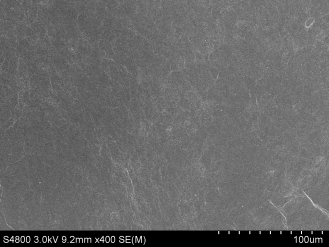


50 μm

20 μm

20 μm


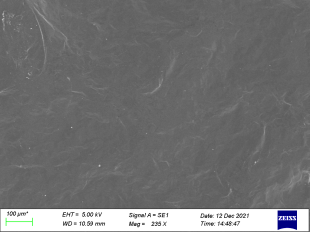




50 μm

20 μm

**Figure S23.** Surface and cross-sectional SEM images of mMAG heat-treated under (a) 270 ^o^C and (b) 3000 ^o^C.

**a b**


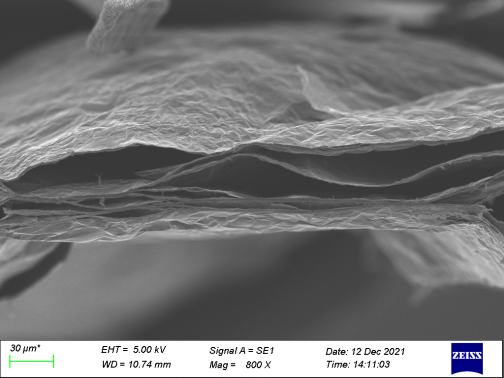

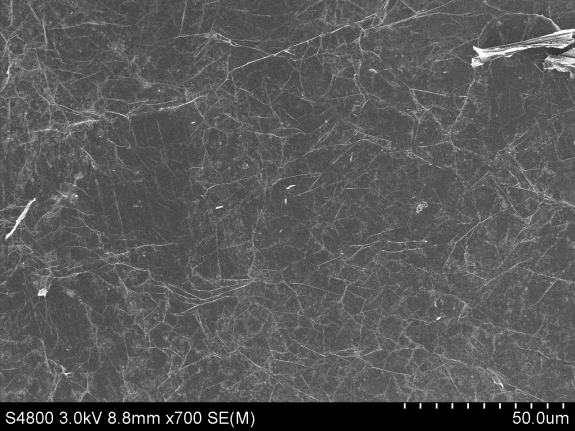

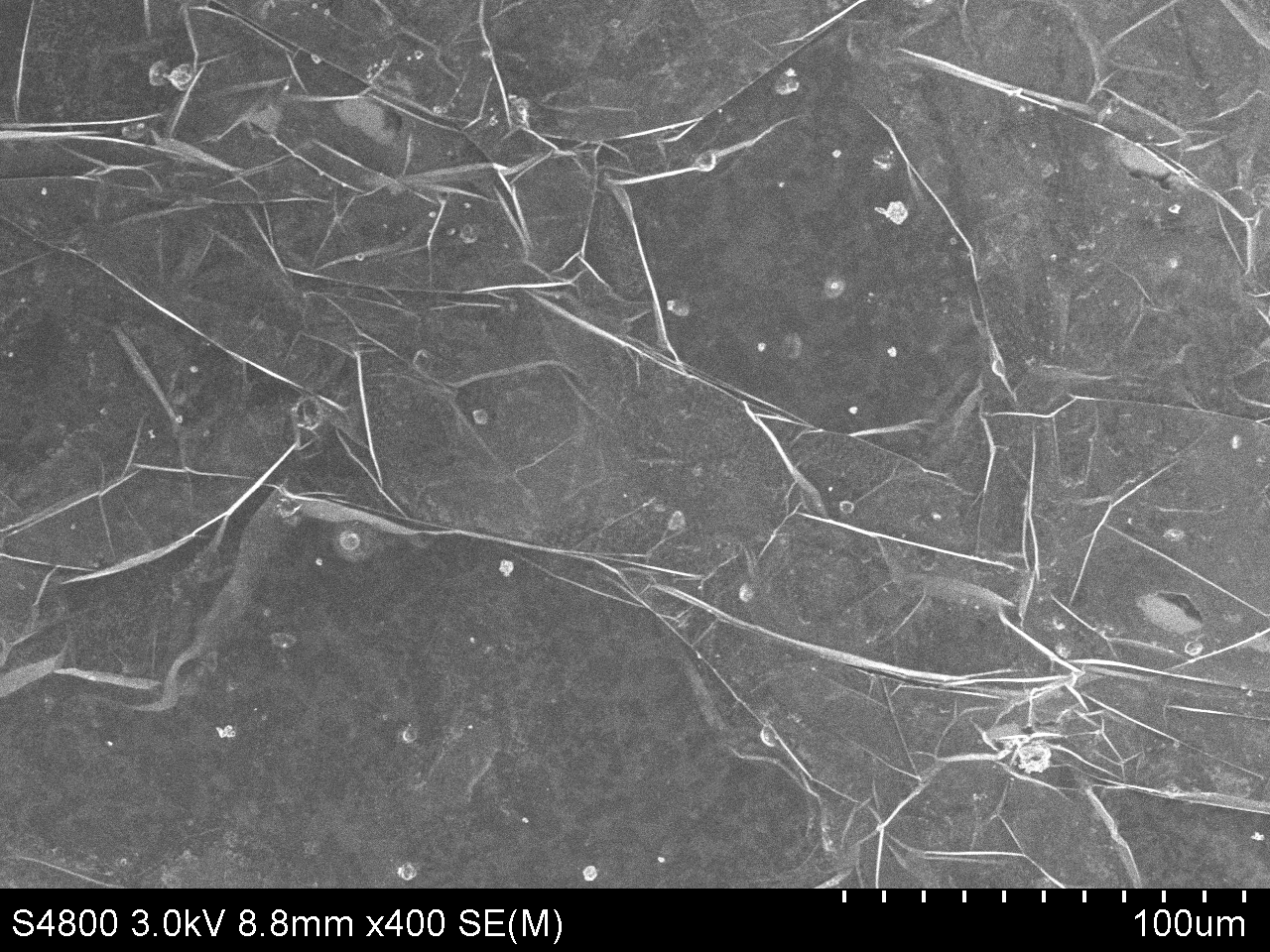


30 μm

6 μm

50 μm

**c**

**Figure S24.** (a) Surface SEM image of commercial artificial PI-based graphite film (GPI). (b) Surface (left) and cross-sectional (right) SEM images of 10 μm-thick GPF (direct scraping of GO/PAN solution). (c) The microfold density of mMAG, GPI, and GPF films.

**Table S1.** The thicknesses of GO/PAN films with different mass ratios after pre-oxidation and heat treatment at 3000 ^o^C.

| GO:PAN (wt%:wt%) | 50%:50% | 40%:60% | 30%:70% |
| --- | --- | --- | --- |
| pre-oxidation | 421 nm | 872 nm | 1590 nm |
| 3000 ^o^C treatment | 243 nm | 400 nm | 594 nm |

**Table S2.** The detachment results in different composite films with 2D sheet and polymer, which is detached (√) or not detached (×).

|  | PAN | Sodium lignin sulfonate |
| --- | --- | --- |
| GO | √ | × |
| rGO | × | √ |

**Table S3.** Electrical conductivity of the graphite film made from high-temperature thermal treatment.

| Material | Thickness (nm) | Conductivity (M S m^-1^) | Temperature (^o^C) | | Ref. |
| --- | --- | --- | --- | --- | --- |
| Carbon Films | 480 | 1.7 | 1400 | 1 | |
| Graphite film | 54 | 1.81 | 2800 | 2 | |
| Graphene film | 48 | 1.8 | 3000 | 3 | |
| Graphite Film | 77 | 0.26 | 1200 | 4 | |
| Graphene film | 10000 | 0.11 | 2800 | 5 | |
| Graphene film | 10000 | 1.06 | 3000 | 6 | |
| Graphite Film | 25000 | 1.1 | 2850 | 7 | |
| Graphene Film | 2700 | 0.1 | 2000 | 8 | |
| This work | 50 | 2.04 | 3000 |  | |

**Table S4.** EMI shielding performances of various EMI materials.

|  | **Materials** | **Thickness**  **[μm]** | **Density**  **[g cm^-3^]** | **SE**  **[dB]** | **SSE/t**  **[dB cm^2^ g^-1^ ]** | **Ref.** |
| --- | --- | --- | --- | --- | --- | --- |
| [**graphene**](about:blank) | Graphite film | 0.231 | 2.25 | 25 | 481000 | 4 |
|  | Graphite film | 0.308 | 2.25 | 26.8 | 386724 | 4 |
|  | Graphite film | 0.385 | 2.25 | 27.8. | 320923 | 4 |
|  | Graphene sheet | 2.8 | 2.14 | 39 | 65087 | 9 |
|  | Graphene film | 31 | 1.63 | 130 | 25727 | 10 |
|  | Graphene film. | 3.4 | 2.03 | 36.8 | 53409 | 11 |
|  | Graphene film | 4 | 1.49 | 38 | 63926 | 12 |
|  | Graphene/CNT | 15 | 1.45 | 57.6 | 26483 | 13 |
|  | Graphene/CNT/PVDF | 100 | 1.77 | 27.58 | 1557 | 14 |
|  | Graphene paper  Graphene aerogel.  Graphene foam  GCF-1400  Reduced graphene oxide fiber | 50  120  300  0.483  30 | 0.67  0.41  0.06  1.63  0.3 | 60  85  25.2  21.72  31 | 17910  17276  14000  275883  33333 | 15  16  17  1  18 |
| **MXene** | Ti_3_C_2_T_x_ | 0.04 | 3.8 | 21 | 1381579 | 19 |
|  | Ti_3_C_2_T_x_ | 0.137 | 3.8 | 33 | 633884 | 19 |
|  | Ti_2_CT_x_ | 0.094 | 3.8 | 13 | 363942 | 19 |
|  | Ti_3_C_2_T_x_ | 1.5 | 2.39 | 45 | 125523 | 20 |
|  | Ti_3_C_2_T_x_ | 11 | 2.39 | 68 | 25853 | 20 |
|  | Ti_3_C_2_T_x_  Mxene/BC film  Ti_3_C_2_T_x_ foam  MXene/Cellulose Paper  MXene Layer/ Cellulose Nanofiber  MXene Foam  Ti3C2Tx MXene/Graphene | 0.94  4  6  24  40  6  1850 | 4.3  3.17  0.39  1.91  1.63  0.39  0.94 | 46  37  32  47  35  32  61 | 120000  29141  136752  2673  7029  136752  11742 | 21  22  23  24  25  26  27 |
| **Metal** | Al foil | 8 | 2.7 | 66 | 30555 | 18 |
|  | Cu foil | 10 | 8.96 | 70 | 7812.5 | 18 |
|  | Cu-wrapped  polymer nanofiber  CuNi-CNT foam  Ag nanowires/PI foam | 1.2 | 1.6 | 45 | 232860 | 28 |
|  |  | 1500 | 0.23 | 54.6 | 1580 | 29 |
|  |  | 5000 | 0.029 | 35 | 2406 | 30 |
| **This Work** | nMAG(PAN, 50%) | 0.05 | 2.0 | 16.19 | 1619000 |  |
|  | nMAG(PAN, 50%) | 0.1 | 2.0 | 25.84 | 1292000 |  |
|  | nMAG(PAN, 50%)  nMAG(PAN, 60%)  nMAG(PAN, 70%) | 0.24  0.4  0.6 | 2.0  2.0  2.0 | 38.69  40.98  48.58 | 806042  512250  404832 |  |

**Supplementary References**

[S1]. M. Tan, D. Chen, Y. Cheng, H. Sun, G. Chen, S. Dong, G. Zhao, B. Sun, S. Wu, W. Zhang, J. Han, W. Han, X. Zhang, Anisotropically oriented carbon films with dual-function of efficient heat dissipation and excellent electromagnetic interference shielding performances. Adv. Funct. Mater. **32**(31), 2202057. (2022). https://doi.org/10.1002/adfm.202202057

[S2]. K. Murashima, Y. Kawashima, S. Ozaki, A. Tatami, M. Tachibana, T. Watanabe, T. Harada, M. Murakami, Modified-edge-support heat treatment method of polyimide for crystalline, large-area, and self-standing ultrathin graphite films. Carbon **181**, 348-357. (2021). <https://doi.org/10.1016/j.carbon.2021.05.036>

[S3]. L. Peng, Y. Han, M. Wang, X. Cao, J. Gao, Y. Liu, X. Chen, B. Wang, B. Wang, C. Zhu, X. Wang, K. Cao, M. Huang, B. V. Cunning, J. Pang, W. Xu, Y. Ying, Z. Xu, W. Fang, Y. Lu, R. S. Ruoff, C. Gao, Multifunctional macroassembled graphene nanofilms with high crystallinity. Adv. Mater*.* **33**(49), 2104195. (2021). <https://doi.org/10.1002/adma.202104195>

[S4]. T. Zhou, C. Xu, H. Liu, Q. Wei, H. Wang, J. Zhang, T. Zhao, Z. Liu, X. Zhang, Y. Zeng, H. Cheng, and W. Ren, Second time-scale synthesis of high-quality graphite films by quenching for effective electromagnetic interference shielding. ACS Nano **14**(3), 3121-3128. (2020). <https://doi.org/10.1021/acsnano.9b08169>

[S5]. J. Ding, O. Rahman, H. Zhao, W. Peng, H. Dou, H. Chen, H. Yu, Hydroxylated graphene-based flexible carbon film with ultrahigh electrical and thermal conductivity. Nanotechnology **28**, 39. (2017). [https://doi.org/](https://doi.org/10.1021/acsnano.9b08169)10.1088/1361-6528/aa8158

[S6]. L. Peng, Z. Xu, Z. Liu, Y. Guo, P. Li, C. Gao, Ultrahigh thermal conductive yet superflexible graphene films. Adv. Mater. **29**(27), 1700589. (2017). https://doi.org/10.1002/adma.201700589

[S7]. R. Song, Q. Wang, B. Mao, Z. Wang, D. Tang, B. Zhang, J. Zhang, C. Liu, D. He, Z. Wu, S. Mu, Flexible graphite films with high conductivity for radio-frequency antennas. Carbon **130**, 164-169. (2018). <https://doi.org/10.1016/j.carbon.2018.01.019>

[S8]. B. Shen, W. Zhai, W. Zheng, Ultrathin flexible graphene film: an excellent thermal conducting material with efficient EMI shielding. Adv. Funct. Mater. **24**(28), 4542-4548. (2014). https://doi.org/10.1002/adfm.201400079

[S9]. S. Wan, Y. Chen, S. Fang, S. Wang, Z. Xu, L. Jiang, R. H. Baughman, Q. Cheng, High-strength scalable graphene sheets by freezing stretch-induced alignment. Nat. Mater. **20**(5), 624-631. (2021). https://doi.org/10.1038/s41563-020-00892-2

[S10]. E. Zhou, J. Xi, Y. Liu, Z. Xu, Y. Guo, L. Peng, W. Gao, J. Ying, Z. Chen and C Gao, Large-area potassium-doped highly conductive graphene films for electromagnetic interference shielding. Nanoscale **9**(47), 18613-18618. (2017). https://doi.org/10.1039/C7NR07030F

[S11]. S. Wan, Y. Chen, Y. Wang, G. Li, G. Wang, L. Liu, J. Zhang, Y. Liu, Z. Xu, A. Tomsia, L. Jiang, Q. Cheng, Ultrastrong graphene films via long-chain π-bridging. Matter **1**(2), 389-401. (2019). https://doi.org/10.1016/j.matt.2019.04.006

[S12]. Q. Wei, S. Pei, X. Qian, H. Liu, Z. Liu, W. Zhang, T. Zhou, Z. Zhang, X. Zhang, H. M. Cheng, W. Ren, Superhigh electromagnetic interference shielding of ultrathin aligned pristine graphene nanosheets film. Adv. Mater. **32**(14), 1907411. (2020). <https://doi.org/10.1002/adma.201907411>

[S13]. B. Zhao, C. Zhao, R. Li, S. M. Hamidinejad, and C. B. Park, Flexible, ultrathin, and high-efficiency electromagnetic shielding properties of poly(vinylidene fluoride)/carbon composite films. ACS Appl. Mater. Interfaces **9**(24), 20873-20884. (2017). https://doi.org/10.1021/acsami.7b04935

[S14]. J. Xu, R. Li, S. Ji, B. Zhao, T. Cui, X. Tan, G. Gou, J. Jian, H. Xu, Y. Qiao, Y. Yang, S. Zhang, and T. Ren, Multifunctional graphene microstructures inspired by honeycomb for ultrahigh performance electromagnetic interference shielding and wearable applications. ACS Nano **15**(5), 8907-8918. (2021). <https://doi.org/10.1021/acsnano.1c01552>

[S15]. .L. Zhang, N. T. Alvarez, M. Zhang, M. Haase, R. Malik, D. Mast, V. Shanov, Preparation and characterization of graphene paper for electromagnetic interference shielding. Carbon **82**, 353-359. (2015). <https://doi.org/10.1016/j.carbon.2014.10.080>

[S16]. J. Xi, Y. Li, E. Zhou, Y. Liu, W. Gao, Y. Guo, J. Ying, Z. Chen, G. Chen, C. Gao, Graphene aerogel films with expansion enhancement effect of high-performance electromagnetic interference shielding. Carbon **135**, 44-51. (2018). <https://doi.org/10.1016/j.carbon.2018.04.041>

[S17]. W. Song, X. Guan, L. Fan, W. Cao, C. Wang, M. Cao, Tuning three-dimensional textures with graphene aerogels for ultra-light flexible graphene/texture composites of effective electromagnetic shielding. Carbon **93**, 151-160. (2015). https://doi.org/10.1016/j.carbon.2015.05.033

[S18]. L. Xu, H. Lu, Y. Zhou, Z. Chi, Z Li, Z. Md, Y. Dong, Y. Fu, Y. Zhu, Q. Ni, Ultrathin, ultralight, and anisotropic ordered reduced graphene oxide fiber electromagnetic interference shielding membrane. Adv. Mater. Technol. **6**(12), 2100531. (2021). <https://doi.org/10.1002/admt.202100531>

[S19]. M. Han, C. E. Shuck, R. Rakhmanov, D. Parchment, B. Anasori, C. M. Koo, G. Friedman and Y. Gogotsi, Beyond Ti_3_C_2_T_x_: MXenes for electromagnetic interference shielding. ACS Nano **14**(4), 5008-5016. (2020). <https://doi.org/10.1021/acsnano.0c01312>

[S20]. F. Shahzad, M. Alhabeb,C. B. Hatter, B. Anasori, S. M. Hong, C. M. Koo, Y. Gogotsi, Electromagnetic interference shielding with 2D transition metal carbides. Science **353**(6304), 1137-1140. (2016). [https://doi.org/](https://doi.org/10.1021/acsnano.0c01312)[10.1126/science.aag2421](https://doi.org/10.1126/science.aag2421)

[S21]. J Zhang, N Kong, S Uzun, A. Levitt, S. Seyedin, P. A. Lynch, S. Qin, M. Han, W. Yang, J. Liu, X. Wang, Y. Gogotsi, J. M. Razal, Scalable Manufacturing of free-standing, strong Ti_3_C_2_T_x_ MXene films with outstanding conductivity. Adv. Mater. **32**(23), 2001093. (2020). <https://doi.org/10.1002/adma.202001093>

[S22]. Y. Wan, P. Xiong, J. Liu, F. Feng, X. Xun, F. M. Gama, Q. Zhang, F. Yao, Z. Yang, H. Luo, Y. Xu, Ultrathin, strong, and highly flexible Ti_3_C_2_T_x_ MXene/bacterial cellulose composite films for high-performance electromagnetic interference shielding. ACS Nano **15**(5), 8439-8449. (2021). <https://doi.org/10.1021/acsnano.0c10666>

[S23]. J. Liu, H. Zhang, R. Sun, Y. Liu, Z. Liu, A. Zhou, Z. Yu, Hydrophobic, flexible, and lightweight MXene foams for high-performance electromagnetic-interference shielding. Adv. Mater. **29**(38), 1702367. (2017). <https://doi.org/10.1002/adma.201702367>

[S24]. W. Cao, F. Chen, Y. Zhu, Y. Zhang, Y. Jiang, M. Ma, F. Chen, Binary strengthening and toughening of MXene/cellulose nanofiber composite paper with nacre-inspired structure and superior electromagnetic interference shielding properties. ACS Nano **12**(5), 4583-4593. (2018). <https://doi.org/10.1021/acsnano.8b00997>

[S25]. B. Zhou, Z. Zhang, Y. Li, G. Han, Y. Feng, B. Wang, D. Zhang, J. Ma, C. Liu, Flexible, robust, and multifunctional electromagnetic interference shielding film with alternating cellulose nanofiber and MXene layers. ACS Appl. Mater. Interfaces **12**(4), 4895-4905. (2020). <https://doi.org/10.1021/acsami.9b19768>

[S26]. J. Liu, H. Zhang, R. Sun, Y. Liu, Z. Liu, A. Zhou, Z. Yu, Hydrophobic, flexible, and lightweight MXene foams for high-performance electromagnetic-interference shielding. Adv. Mater. **29**(38), 1702367. (2017). https://doi.org/10.1002/adma.201702367

[S27]. Y. Chen, X. Zheng, J. Cai, G. Zhao, B. Zhang, Z. Luo, G. Wang, H. Pan, and W. Sun, Sulfur doping triggering enhanced Pt–N coordination in graphitic carbon nitride-supported Pt electrocatalysts toward efficient oxygen reduction reaction. ACS Catal. **12**(12), 7406-7414. (2022). <https://doi.org/10.1021/acscatal.2c00944>

[S28]. Z. Zeng, F. Jiang, Y. Yue, D. Han, L. Lin, S. Zhao, Y. B. Zhao, Z. Pan, C. Li, G. Nyström, J. Wang, Flexible and ultrathin waterproof cellular membranes based on high-conjunction metal-wrapped polymer nanofibers for electromagnetic interference shielding. Adv. Mater. **32**(19), 1908496. (2020). <https://doi.org/10.1002/adma.201908496>

[S29]. K. Ji, H. Zhao, J. Zhang, J. Chen, Z. Dai, Fabrication and electromagnetic interference shielding performance of open-cell foam of a Cu–Ni alloy integrated with CNTs. Appl. Surf. Sci. **311**, 351-356. (2014). <https://doi.org/10.1016/j.apsusc.2014.05.067>

[S30]. J. Ma, K. Wang, M. Zhan, A comparative study of structure and electromagnetic interference shielding performance for silver nanostructure hybrid polyimide foams. RSC Adv. **5**(80), 65283-65296. (2015). https://doi.org/10.1039/C5RA09507G
